# Integrated Multi-Omics Analysis Reveals Key Regulators of Bovine Oocyte Maturation

**DOI:** 10.3390/ijms26093973

**Published:** 2025-04-23

**Authors:** Yassin Kassim, Hao Sheng, Guangjun Xu, Hao Jin, Tariq Iqbal, Mostafa Elashry, Kun Zhang

**Affiliations:** 1 Key Laboratory of Dairy Cow Genetic Improvement and Milk Quality Research of Zhejiang Province, College of Animal Sciences, Zhejiang University, Hangzhou 310058, China; 2 Department of Animal and Poultry Production, Faculty of Agriculture, Minia University, El-Minya 61519, Egypt

**Keywords:** dairy cow, oocyte, metabolomics, transcriptomics, meiosis

## Abstract

A well-regulated metabolism is crucial for optimal oocyte development and embryonic health. However, the metabolic framework governing oocyte maturation remains poorly understood. Using bovine oocytes as a model, we examined metabolomic and transcriptomic alterations during the transition from the germinal vesicle (GV) to the metaphase II (MII) stage. Our findings reveal distinct metabolic shifts, including suppressed β-oxidation combined with the accumulation of long-chain fatty acids (LCFAs). Notably, progesterone emerged as a key regulator of meiotic resumption through its influence on cAMP levels. We also observed enhanced glycolysis, moderate activation of the citric acid cycle (TCA cycle), and suppression of oxidative phosphorylation (OXPHOS), alongside reduced urea cycle flux and shifts in amino acid metabolism favoring glutamate synthesis. Intriguingly, discrepancies between metabolic and transcriptional activities in pathways such as the TCA cycle and nucleotide metabolism suggest asynchronous regulation. These findings provide a comprehensive multi-omics resource, advancing our understanding of the dynamic metabolic and transcriptional landscape during bovine oocyte maturation.

## 1. Introduction

Fertility is fundamentally tied to oocyte quality since the developmental potential of an embryo is dictated mainly by the molecular and cellular activities that occur during oocyte maturation [1,2,3]. This maturation ensures structural and functional readiness for fertilization and embryonic development [4,5]. While in vitro maturation (IVM) of bovine oocytes achieves rates of 80–90%, blastocyst rates rarely exceed 40%, and pregnancy rates are 29–53%, significantly lower than in vivo embryos [6,7,8]. These disparities stem from in vitro culture systems failing to replicate the female reproductive tract’s physiological conditions, hindering optimal oocyte growth, fertilization, and embryo development [9,10].

Understanding these limitations demands a deeper investigation of the molecular processes driving oocyte maturation and embryo development. In this context, metabolomics and transcriptomics serve as useful techniques to examine the complicated metabolic and genetic mechanisms driving oocyte quality [11]. These advanced omics technologies allow researchers to investigate metabolic pathways and gene expression patterns, giving information on important regulators of meiotic progression, energy metabolism, and developmental competence [12,13].

More recently, cutting-edge research has revealed the complex interplay between metabolic pathways and gene expression during oocyte maturation, highlighting the dynamic changes in the metabolism of these pathways, such as glycolysis, oxidative phosphorylation (OXPHOS), amino acid metabolism, and lipid metabolism, based on the specific requirements of oocytes at different developmental stages. For example, studies have shown that mature oocytes in mammals (porcine, mouse, and ovine) primarily metabolize glucose through the TCA cycle and pentose phosphate pathway (PPP) more than glycolysis [12,14,15]. In contrast, during mouse oocyte growth, it was observed that glycolysis is promoted, whereas the TCA cycle is reduced with OXPHOS in growing oocytes [16]. Furthermore, mouse, caprine, and porcine oocytes exhibit a reduction in β-oxidation and a similar trend in the serine–glycine–one-carbon (SGOC) pathway during the GV-to-MII transition, suggesting lipid metabolism may not be the primary energy source at this stage [14,15,17]. Additionally, the supplementation of lipid metabolites, particularly long-chain polyunsaturated fatty acids (PUFAs), has been shown to disrupt meiotic maturation, explaining that it must be downregulated to relieve this inhibitory mechanism [14]. Understanding the complicated interaction between these molecular frameworks is crucial for revealing the processes driving oocyte quality and fertility and developing biomarkers to enhance assisted reproductive technologies (ARTs).

Despite these advances, the metabolic regulation of oocyte maturation remains incompletely understood, particularly in cattle. A major challenge in metabolomic studies is the large sample requirement; for instance, analyzing three developmental stages in mouse oocytes demands ~18,000 oocytes per stage, necessitating a substantial number of mice, about 3200 [14], raising practical concerns for studies in humans or large mammals where oocyte availability is limited. However, recent technological advances now enable ultra-sensitive metabolomic profiling of minute samples: 100 mouse embryos [18], about 125 ovine oocytes or embryos [12], and as few as 8 mice oocytes were analyzed across 6 replicates [16].

In the present study, a dynamic UPLC/MS-based metabolomic profile of bovine oocytes during meiotic maturation was developed. Transcriptome data from a published study were incorporated to support these findings, highlighting the metabolic pathways that promote oocyte development [19]. This comprehensive strategy enabled us to establish a detailed metabolic landscape, revealing multiple distinct metabolic traits associated with the transition from the GV to the MII stage. Collectively, our data provide a robust multi-omics resource and lay the groundwork for identifying new biomarkers to predict and improve oocyte quality.

## 2. Results

### 2.1. Metabolomic and Transcriptomic Profiling of Bovine Oocyte Maturation

**Figure 1:**
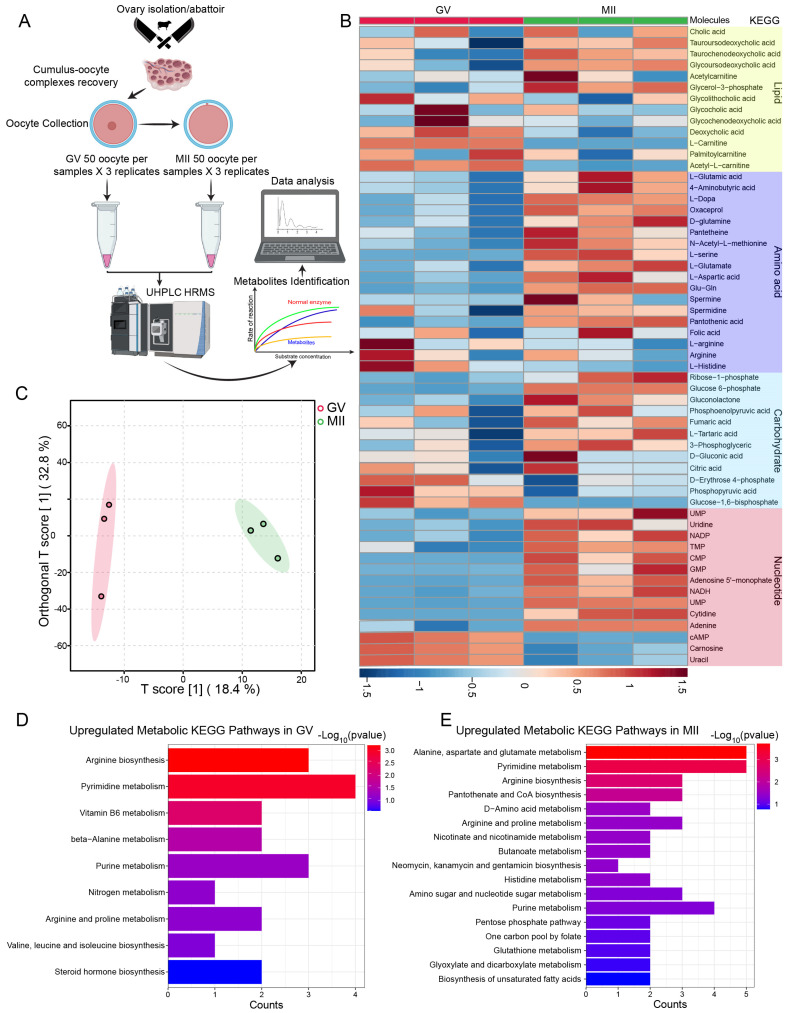
Metabolomic profiling of bovine oocyte maturation. (**A**) Schematic illustration of bovine oocyte isolation at key time points (0 h and 24 h) and workflow for metabolome profiling. (**B**) Heatmap depicting the relative abundance of differential metabolites across metabolic pathways during bovine oocyte maturation. (**C**) PCA plot for nontargeted metabolomics showing clustering of two biological replicates of GV and MII (n = 50 oocytes for each sample). (**D**,**E**) Enriched KEGG pathways in GV and MII based on the upregulated metabolites. See also Supplementary Figure S1 and Table S1.

Earlier research has elucidated several cellular pathways involved in oocyte maturation across different mammalian species [14,15,16,17]. Integrating metabolomic and transcriptomic profiling provides a clearer understanding of how metabolic dynamics regulate meiotic progression in bovine oocytes. This approach offers a comprehensive view of the interplay between gene expression and metabolic changes, revealing key molecular mechanisms underpinning oocyte maturation. In this study, we recovered 300 oocytes from COCs at two key stages: the GV stage and the MII stage (Figure 1A). Using UHPLC-HRMS, we analyzed the intracellular metabolome and identified 95 differential metabolites through *t*-test analysis (*p* < 0.05) and VIP analysis (VIP > 1.00) (Supplementary Figure S1A and Table S1). The alterations in metabolite levels observed during meiotic maturation are summarized in Figure 1B and Supplementary Figure S1B. Robust orthogonal partial least-squares discriminant analysis (OPLS-DA) revealed stage-dependent separation between the two groups (Figure 1C).

Additionally, pathway enrichment analysis of the differential metabolites present at the GV and MII stages (Figure 1D,E, respectively; Supplementary Figure S1C–F) revealed distinct metabolic patterns. Linking the differential metabolomic profiles to the Kyoto Encyclopedia of Genes and Genomes (KEGG) pathways revealed that most amino acids, carbohydrates, and nucleotides increased during meiotic resumption. In contrast, lipid metabolism exhibited significant reductions, particularly β-oxidation and steroid hormone synthesis.

**Figure 2:**
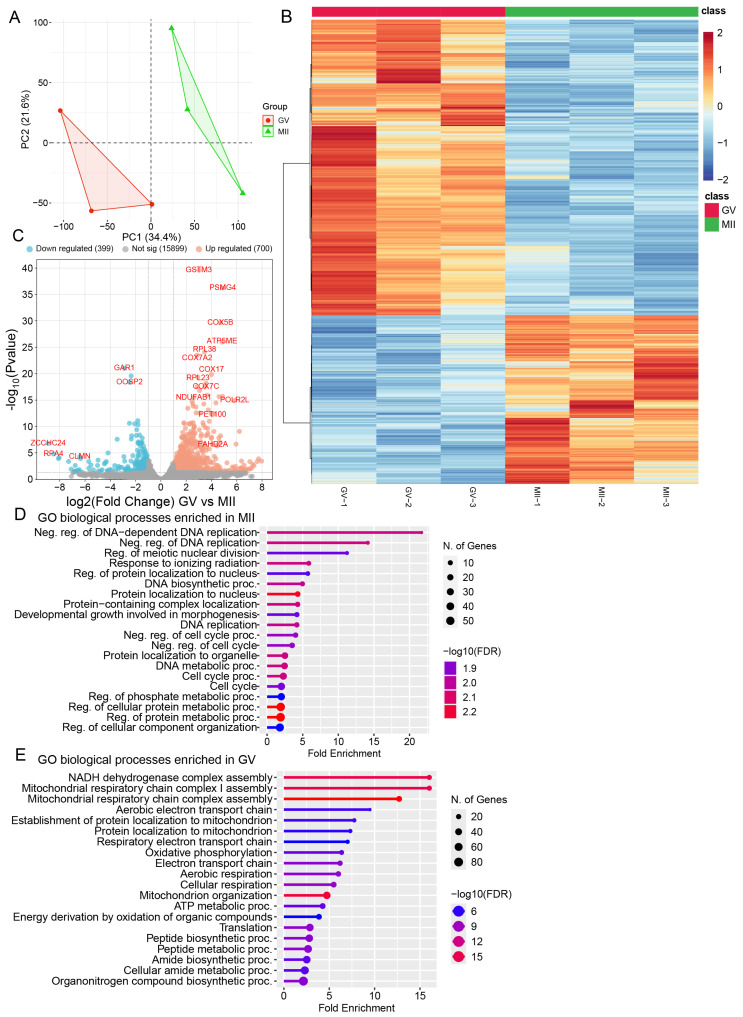
Transcriptomic profiling of bovine oocyte maturation. (**A**) PCA plot for transcriptomic datasets separating 0 h and 24 h oocyte samples. (**B**) Heatmap of hierarchical clustering of 1099 differentially expressed genes (DEGs) from bovine oocytes cultured in vitro at key time points. (**C**) Volcano plot showing the number of upregulated genes (orange dots) or downregulated metabolites (blue dots). The black dotted line indicates the adjusted *p* < 0.05. (**D**) GO biological processes enriched for DEGs in MII-stage oocytes. (**E**) GO biological processes enriched for DEGs in GV-stage oocytes. See also Supplementary Figures S2 and S3.

Gene transcription is a key indicator for predicting and assessing dynamic changes in cellular processes during oocyte development. In our current study, we used previously published transcriptomic data [19] to support and provide additional insights into the metabolomic status of oocytes during maturation (Figure 2). Principal component analysis (PCA) revealed stage-specific separation of the two oocyte groups (Figure 2A). Differential expression analysis identified 16,788 genes, of which 700 were significantly upregulated (adjusted *p* < 0.05, log2(fold change) > 1.5) and 399 were downregulated considerably (adjusted *p* < 0.05, log2(fold change) < 0.67) (Figure 2B,C). Gene ontology (GO) analysis revealed significant enrichment of categories related to metabolic pathways, including OXPHOS, the electron transport chain (ETC), and mitochondrial function, at the GV stage. In contrast, during the MII stage, the most enriched biological processes were associated with DNA replication, cell cycle control, and protein localization (Figure 2D,E).

To enhance our understanding of the metabolic role in oocyte development, we mapped differentially expressed mRNA to the principal KEGG metabolic pathways, revealing multiple significantly altered processes during meiotic maturation (i.e., OXPHOS, ETC, and TCA cycle) (Supplementary Figure S2). Additionally, we conducted enrichment analysis for differentially expressed genes (DEGs) (Supplementary Figure S3). Alternate pathways involving the metabolism of β-oxidation, amino acid, purine, pyrimidine, etc., were uncovered. Collectively, these results offer a comprehensive resource on the metabolomic and transcriptomic processes underlying bovine oocyte maturation. Below, we integrate these findings to study the metabolic alterations throughout meiotic maturation and the potential mechanisms involved.

### 2.2. Lipid Metabolism During Bovine Oocyte Maturation

**Figure 3:**
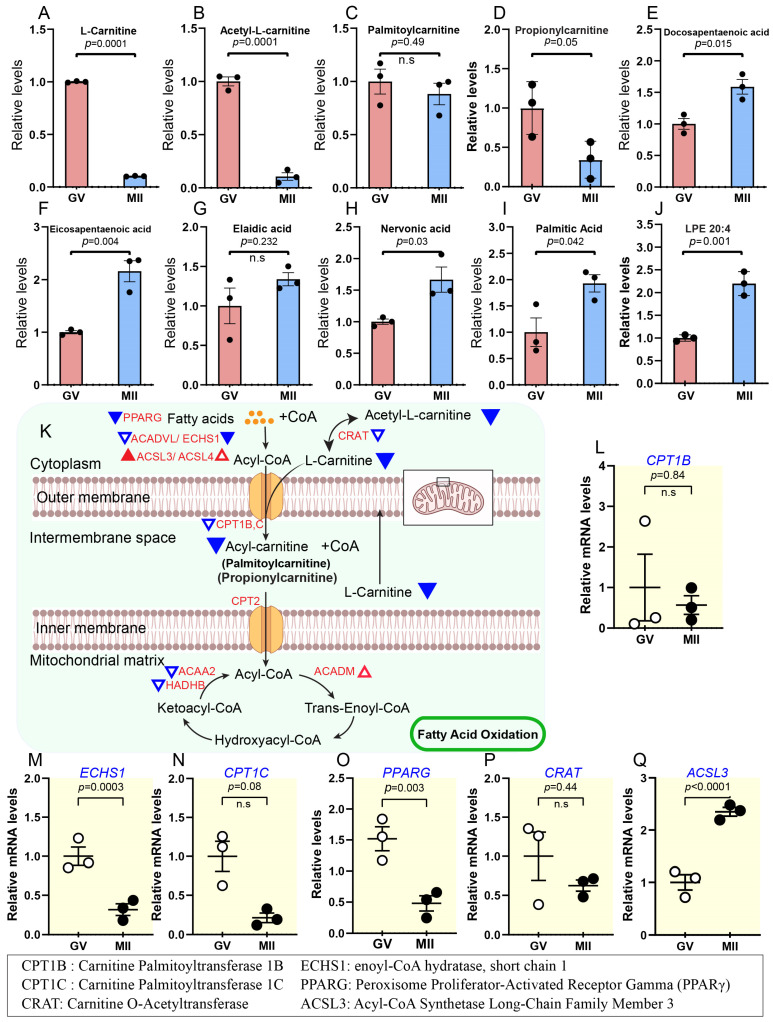
Metabolic features of lipids during oocyte maturation. (**A**–**J**) Relative levels of metabolites associated with lipid metabolism in oocytes at different stages. (**K**) Schematic diagram illustrating the carnitine transport system and fatty acid β-oxidation in mitochondria. Triangles indicate data changes: solid red/blue for significant and hollow red/blue for insignificant. Upward triangles show upregulation, and downward triangles indicate downregulation. Small triangles represent differentially expressed genes (DEGs); larger ones depict metabolite levels, and black and white dots refer to the number of replicates. (**L**–**Q**) Relative expression levels of DEGs in oocytes at various stages are presented. Error bars represent standard deviation (SD). Statistical analysis was performed using Student’s *t*-test. n.s., not significant. See also Supplementary Figures S4 and S5.

**Figure 4:**
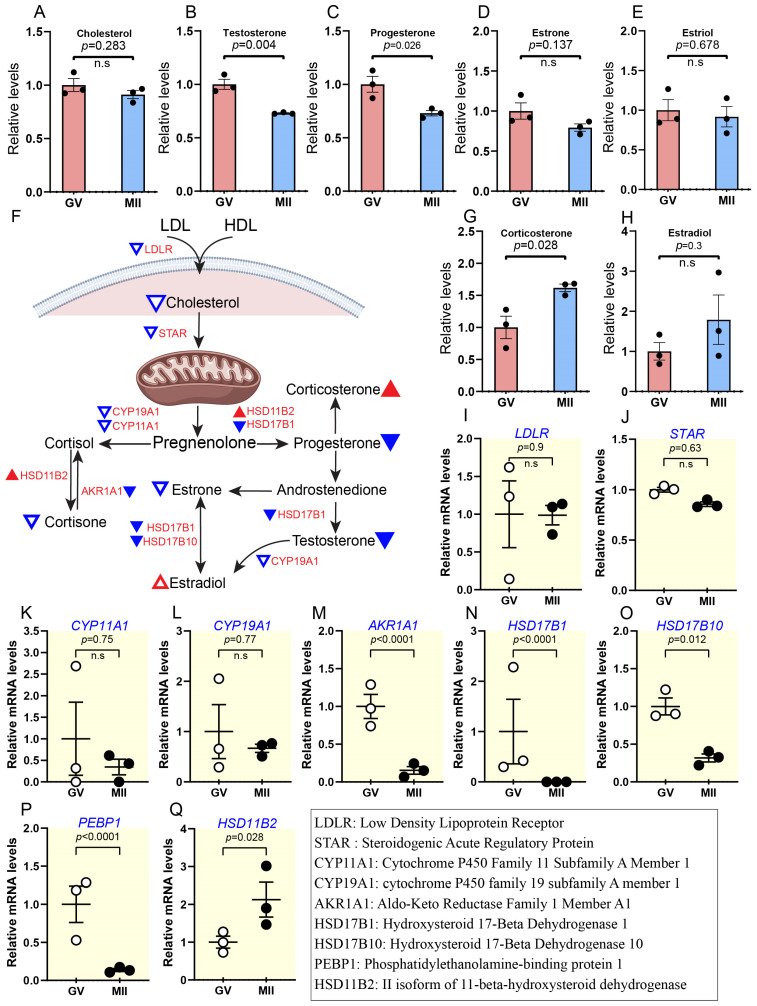
Relative abundance of cholesterol and conversion of steroid hormones during oocyte maturation. (**A**–**H**) Relative levels of metabolites associated with steroid hormones and schematic diagrams illustrating steroid hormone conversion. Triangles indicate data changes: solid red/blue for significant and hollow red/blue for insignificant. Upward triangles show upregulation, and downward triangles indicate downregulation. Small triangles represent differentially expressed genes (DEGs); larger ones depict metabolite levels, and black and white dots refer to the number of replicates. (**I**–**Q**) Relative levels of DEGs related to steroid hormone metabolism during meiotic maturation. Error bars represent standard deviation (SD). Statistical analysis was performed using Student’s *t*-test. n.s., not significant.

From the metabolite map of oocytes (Figure 1B), we observed significant alterations and distinct patterns in lipid metabolism during oocyte maturation. Notably, levels of L-carnitine, acetyl-L-carnitine, and propionylcarnitine key intermediates in β-oxidation along with steroid hormones derived from cholesterol, such as testosterone and progesterone, showed a dramatic decline in MII oocytes compared to the immature GV stage (Figure 3A–D and Figure 4A–C). Conversely, the levels of most detected lipid metabolism-related products (i.e., docosapentaenoic acid, eicosapentaenoic acid, nervonic acid, palmitic acid, arachidonic acid, cholic acid, and glycerol-3-phosphate) increased as oocytes advanced through meiosis (Figure 3E–J; Supplementary Figures S4A, S5 and S6A–F). These findings suggest multifaceted roles for lipid metabolism in oocytes, including supporting nutritional demands, regulating hormone synthesis, and maintaining cellular homeostasis [20,21]. Below, we discuss these roles in detail, emphasizing their contributions to oocyte maturation.

#### 2.2.1. Reduced Fatty Acid β-Oxidation and Accumulation of Long-Chain Fatty Acid During Oocyte Maturation

Fatty acids serve a vital role as substrates for energy production and the synthesis of various lipids [22]. The degradation of long-chain fatty acids (LCFAs) predominantly occurs through mitochondrial fatty acid β-oxidation (FAO). This process begins with the generation of fatty acyl-CoAs on the outer mitochondrial membrane, which is converted into fatty acylcarnitines, such as palmitoylcarnitine, by carnitine palmitoyltransferase I (*CPT1*) when combined with carnitine. Acylcarnitines are transported across the inner mitochondrial membrane via carnitine-acylcarnitine translocase (*CACT*). Within the mitochondrial matrix, carnitine palmitoyltransferase II (*CPT2*) removes the carnitine group, regenerating fatty acyl-CoAs [23]. These fatty acyl-CoAs subsequently enter the β-oxidation pathway to produce ATP, underscoring the essential role of this metabolic process in cellular energy homeostasis [23] (Figure 3K). Although FAO has been implicated as important for maturing mammalian oocytes [14,15] and during follicle activation [16], the metabolic basis underlying this process in bovine oocytes remains unclear.

Notably, our metabolomic analysis revealed a 2- to 9-fold decline in L-carnitine and acetyl-L-carnitine levels, while palmitoylcarnitine content showed no significant change during meiotic resumption (Figure 3A–D). If propionylcarnitine, an odd-chain species produced during amino acid catabolism [24], is considered, it may contribute to increasing the intracellular pool of L-carnitine [25]. Furthermore, we observed an approximately 2-fold increase in the concentrations of various fatty acids, including docosapentaenoic acid (DPA) and eicosapentaenoic acid (EPA) (Figure 3E–J). We speculate that a reduction in β-oxidation activity impairs the breakdown of LCFAs, leading to their accumulation [26]. Consistent with this observation, transcriptomic analysis demonstrated downregulation of key genes involved in lipid metabolism. While *ACSL3*, *ACSL4* and *ACADM* were upregulated, most other genes (e.g., *CPT1B*, *ECHS1*, *CPT1C*, *PPARG*, *CART*) were downregulated during the transition from the GV to MII stage (Figure 3L–Q; Supplementary Figure S4I–Q). These findings collectively indicate that the β-oxidation pathway is suppressed during oocyte maturation in cattle.

#### 2.2.2. Enhanced Fatty Acid Biosynthesis and Their Metabolism During Bovine Oocyte Maturation

Enhanced fatty acid biosynthesis supports the oocyte’s metabolic demands as it prepares for fertilization and early embryonic development [27]. The process involves the upregulation of key enzymes in the fatty acid synthesis pathway, which leads to increased production of LCFAs. These fatty acids are essential for membrane biogenesis, energy storage, and synthesizing bioactive lipids involved in signalling pathways [28]. Along with the LCFAs mentioned above, arachidonic acid (ARA) exhibited dramatic insignificant changes during bovine oocyte maturation (Supplementary Figure S4A).

Notably, ARA, EPA, and DPA are among the most common PUFAs, playing critical roles in cellular metabolism and signalling. These PUFAs act as precursors for eicosanoids (e.g., prostaglandins and leukotrienes), which are pivotal in regulating meiotic progression and oocyte competence [29]. However, their precise functions during bovine oocyte maturation remain unclear and warrant further investigation. Consistent with their elevated levels, we found enrichment of four prostaglandins among seven detected (i.e., prostaglandin A3, prostaglandin D1, prostaglandin E3, and prostaglandin F3α). In contrast, prostaglandin A1, prostaglandin B2, and prostaglandin E1 were downregulated (Supplementary Figure S4B–H). However, just prostaglandin F3α and prostaglandin B2 showed remarkable change (Supplementary Figure S4B,H).

Our metabolomic analysis revealed an 8.2-fold increase in glycerol-3-phosphate (G3P) levels during the transition from the GV to the MII stage (Supplementary Figure S5A). G3P, a key intermediate in glycerophospholipid biosynthesis, serves as the backbone for the synthesis of glycerophospholipids, which are essential components of cellular membranes and signalling pathways (Supplementary Figure S5B). Consistent with this, we observed a significant upregulation in the levels of several glycerophospholipids, including lysophosphatidylethanolamine (LPE) 20:4, lysophosphatidylcholine (LPC) 18:1, phosphatidylcholine (PC) 40:4, and DL-dipalmitoylphosphatidylcholine (DPPC), among others (Supplementary Figure S5C). These findings suggest enhanced glycerophospholipid biosynthesis during oocyte maturation, potentially supporting membrane remodelling, cellular signalling processes, and energy storage as the oocyte prepares for fertilization and early embryonic development. Collectively, the enhanced fatty acid biosynthesis and dynamic changes in PUFAs highlight their critical roles in membrane biogenesis, energy storage, and signalling pathways.

#### 2.2.3. Reduced Cholesterol Levels Coincide with Decreased Steroid Hormone Activity During Maturation

Steroid hormones, derived from cholesterol, play critical roles in regulating oocyte maturation and meiotic progression [15,20]. In our study, we observed a notable decrease in the abundance of steroid hormones by ∼0.7-fold (e.g., progesterone, testosterone, cortisone, and estrone), accompanied by a slight reduction in cholesterol levels in mature oocytes (Figure 4A–E; Supplementary Figure S6I). In contrast, estradiol and corticosterone increased by ∼1.6–1.7 fold (Figure 4G,H). Reducing steroid hormones, particularly progesterone, is crucial for meiotic resumption, as progesterone interacts with natriuretic peptide C (NPPC) to delay germinal vesicle breakdown (GVBD) and maintain oocyte–cumulus communication in cattle [30]. Consistent with this phenomenon, transcriptomic data showed that the accumulation of nine key mRNAs involved in steroidogenesis (*LDLR*, *STAR*, *AKR1A1*, *PEBP1*, *HSD17B10*, etc.) was downregulated (Figure 4I–P). At the same time, *HSD11B2* was upregulated by ∼2.1-fold, from GV to MII (Figure 4Q). Altogether, the results imply that reduced steroid activity might play a crucial role in regulating the meiotic maturation of bovine oocytes.

#### 2.2.4. Elevated Bile Acids and Their Derivatives During Oocyte Maturation

Bile acids (BAs) are the second primary output of cholesterol, alongside steroid hormones, and are classically involved in fat and vitamin absorption in the gut [31,32]. Interestingly, BAs have been identified in follicular fluid (FF) and are associated with embryo development in in vitro fertilization (IVF) settings [33], suggesting their potential involvement in oocyte maturation. However, cholesterol and bile acid metabolism dynamics during oocyte maturation remain poorly understood. The levels of cholic acid and its derivatives, such as glycoursodeoxycholic acid, taurochenodeoxycholic acid, etc., significantly increase upon meiotic resumption (Supplementary Figure S6A–F), In contrast, glycolithocholic acid and deoxycholic acid are downregulated by ∼0.56- and 0.44-fold, respectively (Supplementary Figure S6G,H). This metabolic shift likely reflects cholesterol consumption for the biosynthesis of these bile acid derivatives. These findings underscore the intricate coordination of lipid metabolic pathways required to support meiotic progression and the acquisition of oocyte competence.

### 2.3. Metabolic Characteristics of Carbohydrate Metabolism Activity During Bovine Oocyte Maturation

**Figure 5:**
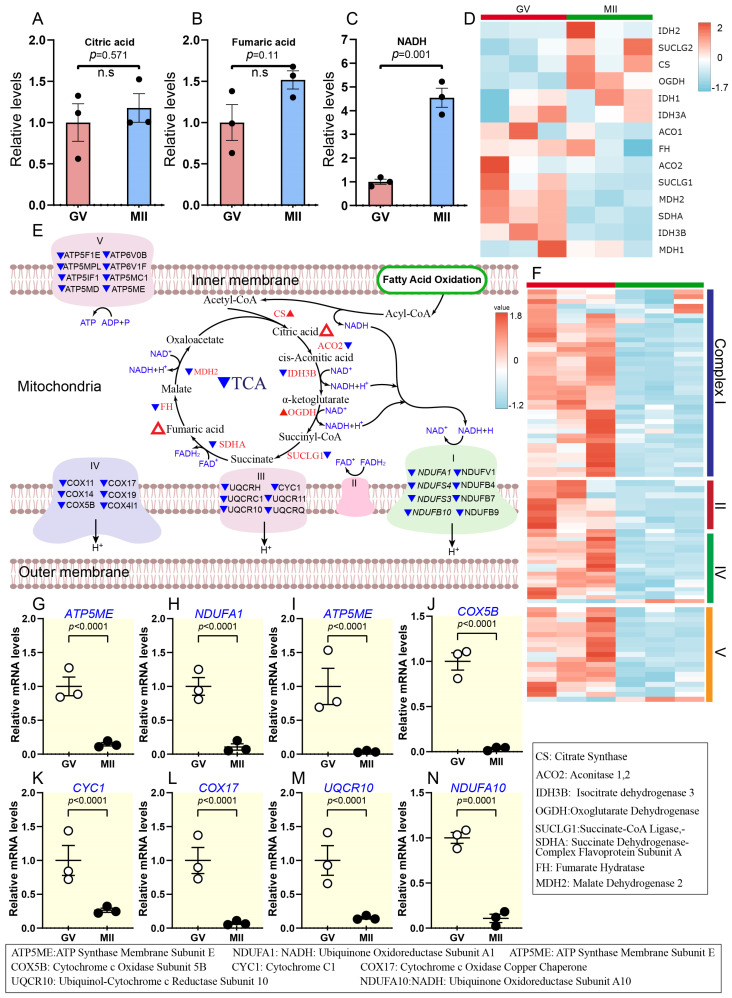
Downregulated oxidative phosphorylation and slightly increased citric acid cycle during oocyte maturation. (**A**–**C**) Relative levels of metabolites associated with the TCA cycle. (**D**) Heatmap illustrating gene expression dynamics related to the TCA cycle in oocytes during maturation. (**E**) Schematic representation of the TCA cycle and oxidative phosphorylation during oocyte maturation. Triangles indicate data changes: solid red/blue for significant and hollow red for insignificant. Upward triangles show upregulation, and downward triangles indicate downregulation. Small triangles represent differentially expressed genes; larger ones depict metabolite levels, and black and white dots refer to the number of replicates. (**F**) Heatmap depicting changes in gene expression associated with OXPHOS during oocyte maturation. (**G**–**N**) Relative abundance of representative genes involved in OXPHOS. Error bars represent standard deviation (SD). Statistical analysis was performed using Student’s *t*-test. n.s., not significant. See also Supplementary Figure S8.

**Figure 6:**
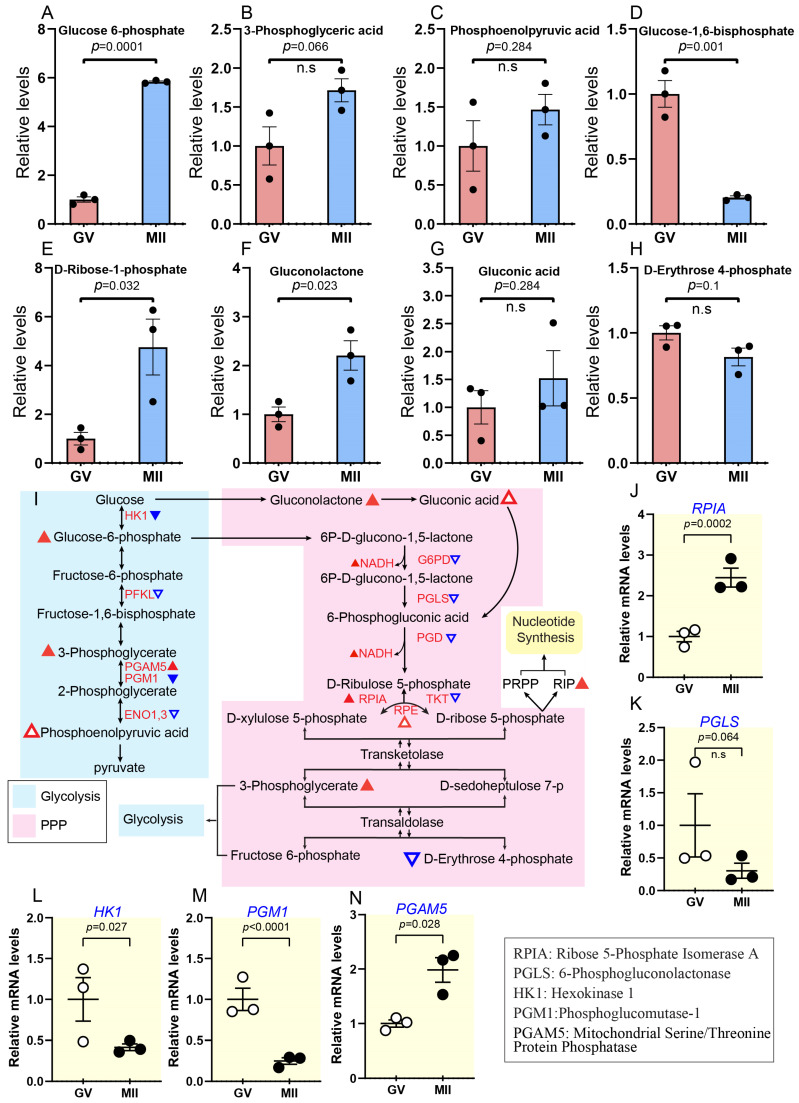
Persistent increase in glycolysis and pentose phosphate pathway during oocyte maturation. (**A**–**H**) Relative levels of metabolites associated with glycolysis and PPP. (**I**) Schematic representation of the glycolysis and PPP during oocyte maturation. Triangles indicate data changes: solid red/blue for significant and hollow red/blue for insignificant. Upward triangles show upregulation, and downward triangles indicate downregulation. Small triangles represent differentially expressed genes; larger ones depict metabolite levels, and black and white dots refer to the number of replicates. (**J**–**N**) Relative abundance of the representative genes involved in PPP and glycolysis. Error bars represent SD. Statistical analysis was conducted using Student’s *t*-test. n.s., not significant. See also Supplementary Figure S7.

Carbohydrate metabolism is critical during oocyte maturation, providing energy and biosynthetic precursors for meiotic progression and developmental competence [15]. Key pathways, including glycolysis, PPP, and TCA, undergo dynamic regulation to meet the metabolic demands of the oocyte along with OXPHOS. Nevertheless, little is known about the dynamics of carbohydrate metabolites during the maturation of bovine oocytes. Temporal metabolome profiles revealed that the number of carbohydrate metabolites increased during oocyte maturation (Figure 5 and Figure 6; Supplementary Figure S7), with enrichment primarily in glycolysis, TCA cycle, PPP, and HBP. Interestingly, transcriptomic analysis indicated a clear decline in OXPHOS activity from the GV to the MII stage (Figure 5).

#### 2.3.1. Activity of Citric Acid Cycle and Inhibition of Oxidative Phosphorylation During Oocyte Maturation

The TCA cycle plays a pivotal role in cellular metabolism by generating high-energy electron carriers essential for OXPHOS [16,34]. It is a central pathway involved in various cellular processes, including the ETC and ATP synthase activity (Complex V). The abundance of two key components of the TCA cycle, citric acid and fumaric acid, showed modest increases during oocyte maturation, with ∼1.2- and ∼1.5-fold increases, respectively. However, these changes were not statistically significant (Figure 5A,B). In addition, NADH-related metabolites, which are critical for ETC function, showed significant elevation (Figure 5C). This simple metabolism-level change is challenging to use as conclusive evidence to judge the increase or decrease of TCA cycle activity. In addition, we found a reduction in six genes at the transcriptional level (*ACO2*, *SUCLG1*, *MDH2*, *SDHA*, *IDH3B*, *MDH2*), while two genes showed an increase (*CS* and *OGDH*) during the GV to MII transition (Figure 5D,E and Supplementary Figure S8).

Notably, the mRNA levels of key genes involved in OXPHOS and the ETC (e.g., *CYC1*, *UQCR10*, *NDUFA1*, *NDUFA10*, *ATP5ME*, *ATP5IF1*, *COX17*, and *COX5B*) were significantly reduced in mature oocytes compared to the GV stage (Figure 5E–N). These findings suggest diminished TCA cycle activity at the transcriptional level and potent inhibition of OXPHOS during meiotic resumption. This result reflects reduced mitochondrial activity in oocytes due to their low ATP demand during arrest as they await fertilization.

#### 2.3.2. Persistent Increase in Glycolysis and Pentose Phosphate Pathway During Oocyte Maturation

Glycolysis degrades glucose to provide ATP and pyruvate to start the TCA cycle, while the PPP generates NADPH and ribose-5-phosphate (R5P) for the biosynthesis of nucleotides and antioxidant defence [35,36]. Collectively, they support cellular energy synthesis, development, and preservation [37,38]. The abundance of three key glycolytic intermediates increased during oocyte maturation: glucose 6-phosphate (G6P) by ~6-fold, 3-phosphoglyceric acid (3PG) by ~2-fold, and phosphoenolpyruvic acid (PEP) by ~1.5-fold (Figure 6A–C). Notable, G6P showed a significant increase. In contrast, glucose-1,6-bisphosphate (G1,6 BP) levels decreased by ~0.2-fold (Figure 6D). Although not directly part of glycolysis, G1,6 BP plays a regulatory role by influencing the activity of phosphofructokinase (PFK), a key enzyme in the glycolytic pathway [39]. At the transcriptional level, only one gene, *PGAM5*, was shown to be upregulated (Figure 6L). In contrast, five genes were downregulated (*PFKL*, *ENO3*, *ENO1*, *HK1*, *PGM1*), and the last two genes showed remarkable changes (Figure 6M,N; Supplementary Figure S7J–L).

Parallel to the glycolytic activity change, the PPP pathway exhibited minimal overall change. Metabolomic analysis revealed an increase in gluconic acid, gluconolactone, and D-Ribose-1-phosphate (R1P) from GV to the MII stage (about ~1.5–2.2-fold), whereas D-erythrose 4-phosphate showed a nonsignificant decrease (Figure 6E–H). R1P is not directly involved in PPP. However, it can be derived from ribose-5-phosphate, a key intermediate in nucleotide metabolism and a crucial starting compound for the enzymatic synthesis of various modified nucleosides like adenosine, guanosine, cytidine, uridine, and deoxythymidine [40] (Figure 6I). Two enzymes involved in this pathway, ribose-5-phosphate isomerase A (RPIA) and ribulose-5-phosphate 3-epimerase (RPE), showed increased mRNA levels during oocyte maturation (Figure 6J; Supplementary Figure S7N). In contrast, the mRNA levels of other PPP-related enzymes, such as *PGLS*, *TKT*, *G6PD*, and *PGD*, showed non-significant decreases during meiotic maturation (Figure 6K; Supplementary Figure S7M–O). Combined metabolomic and transcriptomic analyses suggest that oocytes undergo minimal overall changes in PPP activity, with slight increases in specific intermediates and a gradual strengthening of glycolysis and PPP metabolic patterns as maturation progresses.

#### 2.3.3. Increased Hexosamine Biosynthesis Pathway During Oocyte Maturation

The hexosamine biosynthesis pathway (HBP) is a metabolic system that creates Uridine Diphosphate N-Acetylglucosamine (UDP-GlcNAc), a critical substrate for post-translational modifications and protein glycosylation, which is required for appropriate protein folding, stability, and functioning [41]. Notably, N-linked glycosylation—a process dependent on HBP-derived UDP-GlcNAc—plays a pivotal role in zona pellucida (ZP) structure and sperm binding. Inhibition of N-linked glycosylation disrupts ZP glycoprotein maturation, impairing sperm receptor function and reducing fertilization rates [42,43]. In cumulus cells, most UDP-GlcNAc is converted to hyaluronic acid by hyaluronic acid synthase 2 (HAS2) [44]. Hyaluronic acid, the major structural macromolecule in the extracellular matrix of expanded cumulus cells, accumulates within the COCs during cumulus expansion. This accumulation is a critical step in oocyte maturation, facilitating proper oocyte–cumulus communication and supporting subsequent fertilization [45,46]. However, the underlying mechanisms remain largely unknown.

Our metabolomic data revealed a significant increase in the abundance of three key HBP components during oocyte maturation: UDP-GlcNAc (3.85-fold), N-acetyl-α-D-glucosamine 1-phosphate (GlcNAc-1P; 3.28-fold), and N-acetyl-D-glucosamine (GlcNAc; 2.76-fold) (Supplementary Figure S7A–D). Supporting these findings, transcriptomic analysis showed a trend of elevation in four out of five genes associated with the HBP (*GNPNAT1*, *UAP1*, *GFPT1*, *OGT*) (Supplementary Figure S7E–H), whereas PGM3 was downregulated from GV to MII (Supplementary Figure S7I). Collectively, these data highlight the importance of the HBP in supporting oocyte quality and competence by driving glycosylation mechanisms essential for protein folding, stability, and functional adaptations during maturation, ultimately acquiring the capacity to accept sperm.

### 2.4. Metabolic Characteristics of Amino Acids During Bovine Oocyte Maturation

**Figure 7:**
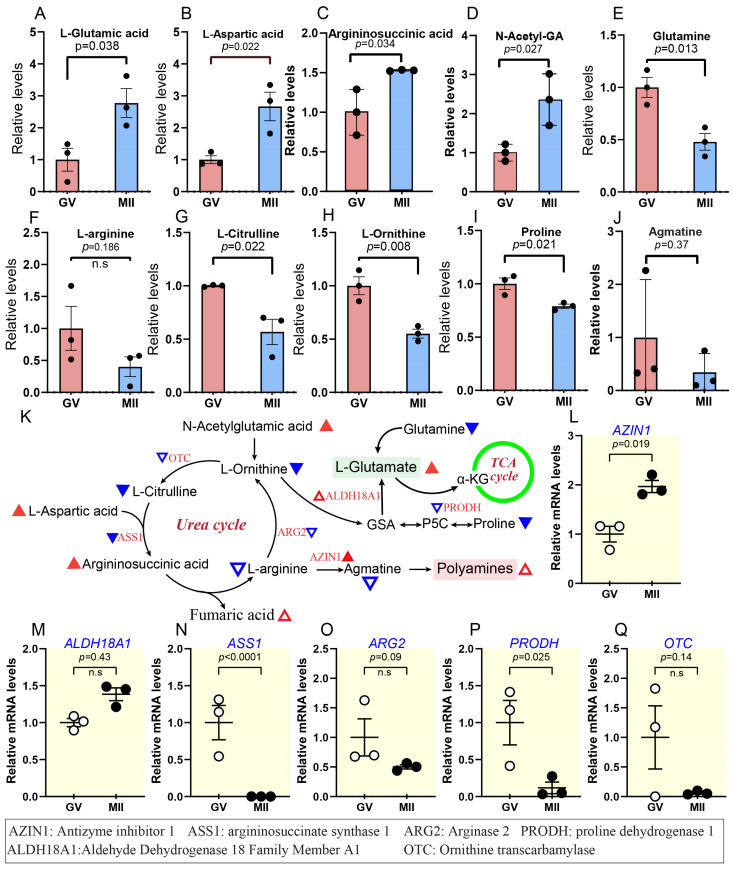
Metabolic characteristics of amino acids and increased glutamic acid synthesis. (**A**–**J**) Relative levels of metabolites associated with glutamic acid synthesis and the urea cycle. (**K**) Schematic representation of the decline in the catabolism of proline and l-arginine and shift to glutamic acid synthesis, green circle refers to the citric acid cycle. Triangles indicate data changes: solid red/blue for significant and hollow red/blue for insignificant. Upward triangles show upregulation, and downward triangles indicate downregulation. Small triangles represent differentially expressed genes; larger ones depict metabolite levels, and black and white dots refer to the number of replicates. (**L**–**Q**) Relative abundance of the representative genes involved in the above pathways. Error bars represent SD. Statistical analysis was conducted using Student’s *t*-test. n.s., not significant. See also Supplementary Figure S9.

Recent studies have elucidated the functions and dynamic changes in amino acids across various species, including mice [14], mares [47], goats [17], and pigs [15] during oocyte maturation. However, the metabolic dynamics of amino acids during bovine oocyte maturation remain largely unexplored. Our temporal metabolite profiles revealed alterations in the level of specific amino acids during oocyte maturation (Figure 7A–D and Supplementary Figure S9A–J). The concentrations of most of the amino acids we detected (e.g., serine, L-glutamate, and L-lysine) significantly increased from GV to matured oocytes. In contrast, some amino acids (e.g., cystine, glutamine, and l-histidine) decreased during this transition (Figure 7E–J and Supplementary Figure S9K–N). This diversity in the amino acid metabolism during oocyte maturation reflects their distinct functional role. Certain amino acids are upregulated to support biosynthetic processes, energy metabolism, and redox balance [48]. Conversely, others are depleted due to their utilization in antioxidant defence, protein synthesis, or specific metabolic pathways essential for maturation [49]. Thus, the variety of metabolic patterns observed in oocytes highlights the intricate and precisely regulated nature of amino acid metabolism.

#### Increased Glutamic Acid Synthesis and Polyamine Metabolism Drive the Catabolism of Arginine and Proline

During oocyte maturation, the metabolic alterations that regulate amino acid catabolism are essential for supporting cellular processes. Specifically, enhanced synthesis of glutamic acid and the upregulation of polyamine metabolism seem to drive the catabolic pathways of arginine and proline, which are essential for oocyte development and meiotic progression [15]. Our metabolomic profiling revealed significant changes in key metabolites during oocyte maturation. Specifically, we observed a 1.5–3.2-fold increase in argininosuccinic acid, L-glutamate, L-aspartic acid, and N-acetyl-glutamic acid (N-Acetyl-GA), which are important intermediates in amino acid metabolism and glutamine/glutamate cycling (Figure 7A–D,K). In contrast, metabolites associated with the urea cycle and arginine metabolism, such as glutamine, L-arginine, L-citrulline, L-ornithine, proline, and agmatine, showed a 0.78–0.37-fold decrease as oocytes matured (Figure 7E–K).

Meanwhile, transcriptome analysis revealed a decrease in the mRNA levels of four out of six genes (*OTC*, *ARG2*, *ASS1*, and *PRODH*) involved in the metabolic pathways mentioned above, while the mRNA levels of *ALDH18A1* and *AZIN1* were upregulated (Figure 7L–Q). Glutamate plays a multifaceted role beyond its involvement in protein synthesis. It is integral to many biological processes, such as taking in nitrogen, making nucleosides, amino acids, and cofactors, and starting the citric cycle over (Walker & van der Donk, 2016). These findings suggest a reduction in the flux through the urea cycle and a shift in amino acid metabolism that supports glutamate synthesis, which may be important during the cell’s transition to a fully mature state [50].

### 2.5. Progressive Increase in Nucleotide Synthesis During Oocyte Maturation

Nucleotide biosynthesis occurs via two pathways: the de novo process, which generates nucleotides from tiny precursor molecules, and the salvage pathway, which reuses existing purine or pyrimidine bases and nucleosides. In de novo purine synthesis, ribose 5-phosphate is converted into phosphoribosyl pyrophosphate (PRPP), resulting in the formation of inosine monophosphate (IMP), which serves as a precursor for adenosine monophosphate (AMP) and guanosine monophosphate (GMP). Likewise, de novo pyrimidine synthesis culminates in the production of uridine monophosphate (UMP), which acts as the precursor for further pyrimidine nucleotides [15,51].

**Figure 8:**
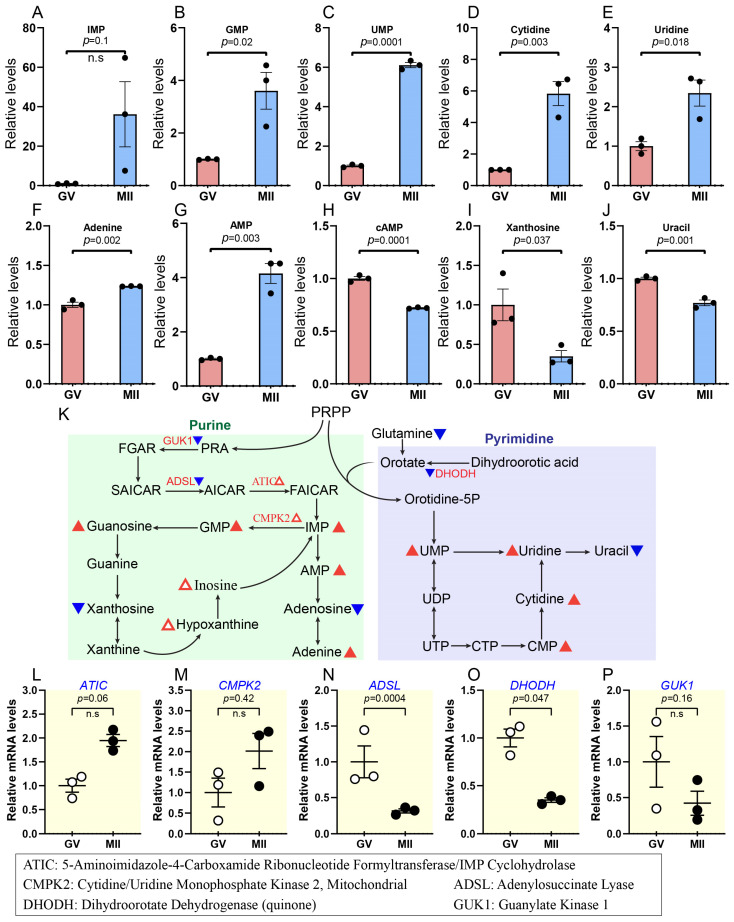
Nucleotide metabolism alterations during bovine oocyte maturation. (**A**–**J**) Relative levels of metabolites related to nucleotide metabolism in oocytes at different stages. (**K**) Schematic diagram of purine and pyrimidine metabolism during oocyte maturation. Triangles indicate data changes: solid red/blue for significant and hollow red for insignificant. Upward triangles show upregulation, and downward triangles indicate downregulation. Small triangles represent differentially expressed genes; larger ones depict metabolite levels, and black and white dots refer to the number of replicates. (**L**–**P**) Relative abundance of the representative genes involved in purine and pyrimidine metabolism. Error bars represent SD. Statistical analysis was conducted using Student’s *t*-test. n.s., not significant. See also Supplementary Figure S10.

Our metabolomic analysis revealed that the concentrations of several key metabolites involved in nucleotide synthesis exhibited a significant increase (i.e., IMP: ~36-fold; GMP: ~3.6-fold; UMP: ~6-fold; cytidine: ~5.8-fold; and uridine: ~2.3-fold) in oocytes transitioning from GV to MII (Figure 8A–G and Supplementary Figure S10A–F). In contrast, numerous nucleotides, such as AMP, cyclic adenosine monophosphate (cAMP), deoxythymidine diphosphate (DTDP), adenosine diphosphate (ADP), and adenosine, showed a decline during this transition (Figure 8G–J; Supplementary Figure S10G–L). In parallel, transcriptomic data indicated a modest overexpression of two genes involved in the de novo nucleotide synthesis pathway: *CMPK2* and *ATIC* (Figure 8L,M). However, the expression of most genes associated with this pathway (e.g., *ADSL*, *GUK1*, *DHODH*, *DUT*, and *GMPR*) decreased throughout oocyte maturation (Figure 8N–P; Supplementary Figure S10M–T). The slight change in transcription observed may be attributed to the naturally reduced transcription activity during oocyte maturation, as mentioned recently [52]. This reduction may be key in preparing the oocyte for fertilization and early embryogenesis. These data indicate that nucleotide metabolism pathways are important in mechanisms regulating meiotic maturation. In addition, the oocyte accumulates large stores of maternal mRNA, which is crucial for early embryonic development before the zygotic genome is activated.

## 3. Discussion

Oocyte maturation is a complex process regulated at multiple molecular levels. Using bovine oocytes at the GV and MII stages, we applied advanced MS-based metabolomics to analyze global metabolic changes during maturation. By integrating these data with published transcriptomic findings [19], we identified key metabolic pathways, including (a) suppressed β-oxidation, (b) enhanced fatty acid biosynthesis, (c) enhanced glycolysis with moderate activation of the TCA cycle, (d) suppression of oxidative phosphorylation, (d) a shift in amino acid metabolism favoring glutamate synthesis, (e) reduced urea cycle flux and (f) enhanced purine synthesis and pyrimidine synthesis. This comprehensive multi-omics analysis deepens our understanding of metabolic and transcriptional dynamics in bovine oocyte maturation, providing insights for prediction in biomarkers of oocyte quality and improving in vitro culture systems for assisted reproduction.

Among the metabolic pathways identified, lipid metabolism exhibited notable changes during oocyte maturation. Specifically, β-oxidation declined in mature oocytes, as evidenced by decreased levels of L-carnitine, acetyl-L-carnitine, and propionylcarnitine, along with palmitoylcarnitine, which showed a non-significant decrease (Figure 3A–D). This finding aligns with observations in porcine oocytes, which also exhibit suppression of β-oxidation during maturation [15]. A decline in β-oxidation may lead to the accumulation of LCFAs that are not processed into acetyl-CoA for energy production. Our metabolomic analysis revealed that numerous LCFAs, such as ARA, EPA, nervonic acid, palmitic acid, and DPA (Figure 3E–J; Supplementary Figures S4A and S5), exhibited elevated changes during bovine oocyte maturation. This buildup can promote lipid droplet formation as cells store the excess fatty acids to prevent lipotoxicity [53,54,55]. In contrast, such metabolic imbalances may contribute to the lower developmental efficiency observed in oocytes produced in vitro compared to those developed in vivo [56,57].

Steroid hormones were demonstrated to decrease with a slight decrease in cholesterol; their high levels can inhibit meiotic progression in bovine oocytes, while their reduction allows for GVBD and maturation [30]. Additionally, the decrease in cholesterol may play a role in promoting MII arrest [14]. It was discovered that progesterone interacts with NPPC to delay GVBD and enhance oocyte–cumulus communication in cattle; also, in human oocytes, it can affect cAMP levels, which regulate meiotic arrest and resumption. Consistent with this, we found that cAMP levels decrease in mature oocytes (Figure 8H). Reducing steroid hormone signalling can allow for the decrease in cAMP levels required for meiotic progression [58]. At the transcriptomic level, several genes exhibited decline during maturation, like *AKR1A1*, *PEBP1*, *HSD17B10*, etc. (Figure 4I–P).

While metabolic and transcriptional changes often align to regulate oocyte maturation, our study also revealed intriguing discrepancies between these two levels of regulation in certain pathways. For instance, in the TCA cycle, metabolic activity slightly increased (Figure 5A,B), while transcriptional activity decreased. Most genes, including *FH*, *IDH3B*, and *SUCLG1*, were downregulated, with only *OGDH* showing a significant increase (Figure 5D,E; Supplementary Figure S8). This trend was also evident in nucleotide metabolism, where most nucleotides increased in developed oocytes (Figure 8A–G, Supplementary Figure S10A–F). Meanwhile, the expression of essential genes related to nucleotide metabolism exhibited a significant decline during the transition from GV to MII (Figure 8N–P; Supplementary Figure S10M–T).

These discrepancies between metabolic and transcriptional activity may be explained by the unique regulatory mechanisms governing oocyte maturation. Our findings highlight that transcriptomic and metabolomic changes do not necessarily occur simultaneously. Specifically, during oocyte growth, the oocyte accumulates substantial amounts of maternal mRNAs, proteins, and metabolites, including nucleotides and enzymes, for their synthesis [44,59,60]. As maturation progresses, the oocyte relies more on these pre-stored resources rather than de novo transcription and translation. This could explain why nucleotide levels remain high even as the expression of nucleotide metabolism genes decreases.

Furthermore, transcription drastically declines toward the end of growth and becomes undetectable during meiotic maturation [61,62,63]. Moreover, increased metabolism of the PPP and purine–pyrimidine metabolism may assist the creation of raw materials for significant DNA and RNA synthesis during early embryonic development [12]. Post-transcriptional gene regulation, including mRNA modifications, may further explain this disconnect between transcriptional and metabolic activity. These regulatory mechanisms ensure maternal mRNAs’ stability, localization, and translation efficiency, allowing metabolic pathways to remain active even when transcription is silenced. Interestingly, we observed an increase in the HBP, which is closely associated with protein glycosylation and may contribute to post-transcriptional regulation by influencing mRNA stability and translation (Supplementary Figure S7A–D). However, as this is a broad study area, we do not delve deeply into this topic [64,65].

In addition, the slight activity of the TCA cycle was accompanied by low transcriptomic activity of OXPHOS during the transition from the GV to the MII stage (Figure 5E–N). Based on these integrated profiles, we provide insights into the metabolic and transcriptomic mechanisms underlying the decline in OXPHOS activity during the transition from the GV to the MII stage in bovine oocytes [66]. Our findings in this area, similar to those observed in sheep oocytes, are characterized by a decreased TCA cycle, oxidative phosphorylation, and ATP content [12]. OXPHOS inhibition reduces reactive oxygen species (ROS) generation by limiting electron flow through the mitochondrial ETC. ROS, as a byproduct of mitochondrial complex I activity, plays a dual role in cellular physiology. While moderate levels of ROS are essential for signalling [67,68], excessive accumulation can lead to oxidative damage, affecting DNA, proteins, and lipids [69]. This damage compromises oocyte quality and developmental potential. Consequently, reduced OXPHOS helps mitigate oxidative stress, preserving cellular integrity for subsequent development. In addition, a decline in ATP levels during oocyte maturation is a well-documented phenomenon crucial for preparing the oocyte for fertilization [70]. Another phenomenon reported by Dalton et al. [71] is that ATP was consumed faster by oocytes undergoing maturation (MI) and at the MII stage than in oocytes arrested at the GV stage. While ATP is essential for processes like spindle assembly, chromosomal segregation, and cytoskeletal organization, its controlled reduction may reflect a metabolic shift to optimize energy utilization for subsequent embryonic development. Consistent with this metabolic finding, transcriptomic analysis revealed an increase in the ETC and OXPHOS at GV stage compared to MII (Figure 2E).

Along with dynamic changes in the metabolism of lipids and carbohydrates, during oocyte maturation, considerable modifications occurred in amino acid metabolism, indicating that dynamic metabolic reprogramming is essential for developmental competence. Changes in protein metabolism increase glutamic acid production, and accelerated polyamine metabolism promotes the catabolism of arginine and proline (Figure 7A–J and Supplementary Figure S9F,G). This metabolic change may serve numerous roles, such as maintaining cellular signalling, redox equilibrium, and biosynthetic activities required for development [15].

The observed drop in arginine and proline levels may be connected to their consumption in polyamine production, a mechanism required for cell proliferation and differentiation. Polyamines, such as putrescine, spermidine, and spermine, are generated from arginine and serve critical roles in chromatin remodelling, RNA stability, and oxidative stress control [72,73,74]. Meanwhile, proline on the other hand, contributes to energy production and the creation of intermediates that feed into the TCA cycle [75]. The concomitant drop in urea cycle activity fits with lower arginine, l-ornithine, and l-citrulline availability since they are critical substrates for this pathway (Figure 7K). This decline may also indicate the oocyte’s need to preserve energy and nitrogen, reallocating resources toward activities that promote maturation and later embryonic development. Notably, urea supplementation in maturation media significantly disrupted the amino acid cycle in bovine COCs, CCs, and denuded oocytes (DOs), primarily due to the depletion of essential amino acids required for proper development and maturation [76]. These results emphasize the delicate balance between amino acid metabolism and the metabolic needs of the oocyte during maturation.

A strength of our research is using bovine oocytes to study oocyte maturation’s metabolomic and transcriptomic landscapes. Human and cattle share key similarities in oocyte maturation, including reproductive strategies, metabolic pathways, and physiology [77,78], making bovine oocytes a more relevant model for human studies than rodents or other mammals. However, the entire understanding of oocytes cannot be isolated from their surrounding cumulus cells (CCs). The metabolic co-dependence of oocytes and CCs is essential for their functional regulation and facilitation of oocyte maturation [79,80]. Consequently, metabolomic analysis of cumulus cells is crucial for understanding the metabolic network regulating oocyte growth. In addition, three important questions must be covered in future studies: (a) How do oocytes and cumulus cells mutually regulate metabolic pathways during maturation? (b) How does oocyte plasticity adapt to metabolome pathway suppression during maturation and subsequent embryo development? (c) What is the relationship between metabolic changes during bovine oocyte maturation and epigenetic modifications?

## 4. Materials and Methods

### 4.1. Bovine Oocyte Maturation and Sample Collection

In vitro maturation (IVM) was conducted in accordance with the procedures that have been previously published [81]. Briefly, bovine ovaries were obtained from a local slaughterhouse and transferred to the laboratory in saline supplemented with 200 IU/mL penicillin (P7794, Sigma, St. Louis, MO, USA) and 200 IU/mL streptomycin (S1277, Sigma, St. Louis, MO, USA) at 30–35 °C within 2–4 h. After the ovaries were washed three times in saline, cumulus–oocyte complexes (COCs) possessing at least three layers of cumulus cells (CCs) were retrieved from 3 to 8 mm follicles at the surface of the ovaries. Subsequently, COCs displaying evenly granulated cytoplasm and at least three layers of compacted CCs were transferred to an IVM medium composed of Medium-199 (M4530) supplemented with 10% fetal bovine serum (Gibco-BRL, Grand Island, NY, USA), 1 IU/mL follicle-stimulating hormone (Sansheng Biological Technology, Ningbo, China), 0.1 IU/mL luteinizing hormone (Solarbio, Beijing, China), 1 mM sodium pyruvate (Thermo Fisher Scientific, Waltham, MA, USA), 2.5 mM GlutaMAX (Thermo Fisher Scientific, Waltham, MA, USA), and 10 μg/mL gentamicin at 38.5 °C in an atmosphere of 5% CO_2_ and humidified air for 22–24 h. To collect GV stage oocytes, COCs were denuded immediately after retrieval (0 h of IVM) by transferring them into a preheated medium containing 0.3% (*w*/*v*) hyaluronidase (H3506, Sigma-Aldrich) at 38.5 °C for 5 min. For MII-stage oocytes, COCs were denuded after 24 h of maturation, and the extrusion of the first polar body was used as a marker of nuclear maturation.

### 4.2. Metabolomic Analysis

#### 4.2.1. Metabolomic Sample Collection

GV and MII oocytes were harvested separately (50 oocytes per sample, 3 samples for each stage), placed in 1.5 mL Eppendorf tubes with minimal liquid, snap-frozen in liquid nitrogen, and kept at −80 °C. For metabolite extraction, the samples (100 μL) were placed in the EP tubes and resuspended with prechilled 80% methanol by a well vortex. Then, the samples (100 μL) were placed in the EP tubes and resuspended with prechilled 80% methanol by well vortex. Then, the samples were incubated on ice for 5 min and centrifuged at 15,000× *g*, 4 °C for 20 min. Some of the supernatant was diluted to a final concentration containing 53% methanol by LC-MS grade water. The samples were subsequently transferred to a fresh Eppendorf tube and then were centrifuged at 15,000× *g*, 4 °C for 20 min. Finally, the supernatant was injected into the LC-MS/MS system analysis [82,83].

#### 4.2.2. UHPLC-MS/MS Analyses

UHPLC-MS/MS analyses were performed using a Vanquish UHPLC system (Thermo Fisher, Karlsruhe, Germany) coupled with an Orbitrap Q ExactiveTM HF mass spectrometer or Orbitrap Q ExactiveTMHF-X mass spectrometer (Thermo Fisher, Karlsruhe, Germany) in Novogene Co., Ltd. (Beijing, China). Samples were injected onto a Hypersil Goldcolumn (100 × 2.1 mm, 1.9 μm) using a 12 min linear gradient at a flow rate of 0.2 mL/min. The eluents for the positive and negative polarity modes were eluent A (0.1% FA in Water) and eluent B (Methanol). The solvent gradient was set as follows: 2% B, 1.5 min; 2–85% B, 3 min; 85–100% B, 10 min; 100–2% B, 10.1 min; 2% B, 12 min. A Q ExactiveTM HF mass spectrometer was operated in positive/negative polarity mode with a spray voltage of 3.5 kV, capillary temperature of 320 °C, sheath gas flow rate of 35 psi and aux gas flow rate of 10 L/min, S-lens RF level of 60 and Aux gas heater temperature of 350 °C.

#### 4.2.3. Metabolomic Data Analysis

The raw data files generated by UHPLC-MS/MS were processed using the Compound Discoverer 3.3 (CD3.3, Thermo Fisher) to perform peak alignment, peak picking, and quantitation for each metabolite. The main parameters were set as follows: peak area was corrected with the first QC; actual mass tolerance, 5 ppm; signal intensity tolerance, 30%; and minimum intensity, etc. After that, peak intensities were normalized to the total spectral intensity. The normalized data were used to predict the molecular formula based on additive ions, molecular ion peaks, and fragment ions. And then the peaks were matched with the mzCloud (https://www.mzcloud.org/, accessed on 14 April 2025), mzVault, and MassList databases to obtain the accurate qualitative and relative quantitative results. Statistical analyses were performed using the statistical software R (R version R-3.4.3), Python (Python 2.7.6 version)and CentOS (CentOS release 6.6), When data were not normally distributed, they were standardized according to the formula sample raw quantitation value/(The sum of sample metabolite quantitation value/The sum of QC1 sample metabolite quantitation value) to obtain relative peak areas; and compounds whose CVs of relative peak areas in QC samples were greater than 30% were removed, and finally the metabolites’ identification and relative quantification results were obtained. The variable importance in projection (VIP) value > 1.00 and *p* value < 0.05 of each metabolite were used as the combined cut-offs of the statistical significance. The integration and visualization of metabolomic and transcriptomic data were performed using KEGG Mapper (v4.1) (https://www.genome.jp/kegg/ and http://ge-lab.org/go/, accessed on 14 April 2025) and used for enrichment analysis of transcriptomic data. Metabolomic analysis was conducted using the online software MetaboAnalyst (https://www.metaboanalyst.ca and https://www.bioinformatics.com.cn, accessed on 14 April 2025).

### 4.3. Statistical Analysis

All statistical analyses were conducted using GraphPad Prism software (v8.0 for Windows). Data are presented as mean ± standard deviation (SD) unless otherwise specified. Student’s *t*-test was employed to determine significant differences between groups. A *p*-value of <0.05 was considered statistically significant.

## 5. Conclusions

In summary, by integrating metabolomic and transcriptomic analyses, we have uncovered a comprehensive global picture of the metabolic characteristics underlying bovine oocyte maturation. Our dataset provides valuable insights into the metabolic processes driving meiotic progression in oocytes. These findings will pave the way for novel approaches to manipulate the identified metabolic pathways, ultimately improving oocyte quality, consequently embryonic development, and advancing optimized in vitro culture systems.

## Data Availability

The data supporting the findings of this study are available within the article and its Supplementary Materials.

## Supplementary Materials

The following supporting information can be downloaded at: https://www.mdpi.com/article/10.3390/ijms26093973/s1.

## Abbreviations

GV	Germinal Vesicle
MII	Metaphase II
IVM	In Vitro Maturation
COCs	Cumulus–Oocyte Complexes
LCFAs	Long-Chain Fatty Acids
TCA Cycle	Citric Acid Cycle (Tricarboxylic Acid Cycle)
OXPHOS	Oxidative Phosphorylation
PPP	Pentose Phosphate Pathway
SGOC	Serine–Glycine–One-Carbon
PUFAs	Polyunsaturated Fatty Acids
ART	Assisted Reproductive Technologies
UHPLC-HRMS	Ultra-High-Performance Liquid Chromatography–High-Resolution Mass Spectrometry
OPLS-DA	Orthogonal Partial Least-Squares Discriminant Analysis
KEGG	Kyoto Encyclopedia of Genes and Genomes
GO	Gene Ontology
DEGs	Differentially Expressed Genes
FAO	Fatty Acid β-Oxidation
CPT1	Carnitine Palmitoyltransferase I
CACT	Carnitine-Acylcarnitine Translocase
CPT2	Carnitine Palmitoyltransferase II
ARA	Arachidonic Acid
EPA	Eicosapentaenoic Acid
PEP	Phosphoenolpyruvic acid
PFK	Phosphofructokinase
DPA	Docosapentaenoic Acid
G3P	Glycerol-3-Phosphate
LPE 20:4	Lysophosphatidylethanolamine
LPC 18:1	Lysophosphatidylcholine
PC 40:4	Phosphatidylcholine
DPPC	DL-Dipalmitoylphosphatidylcholine
NPPC	Natriuretic Peptide C
GVBD	Germinal Vesicle Breakdown
cAMP	Cyclic Adenosine Monophosphate
BAs	Bile Acids
FF	Follicular Fluid
IVF	In Vitro Fertilization
HBP	Hexosamine Biosynthesis Pathway
UDP-GlcNAc	Uridine Diphosphate N-Acetylglucosamine
GlcNAc-1P	N-Acetyl-α-D-Glucosamine 1-Phosphate
GlcNAc	N-Acetyl-D-Glucosamine
HAS2	Hyaluronic Acid Synthase 2
ROS	Reactive Oxygen Species
ETC	Electron Transport Chain
R5P	Ribose-5-Phosphate
R1P	D-Ribose-1-Phosphate
RPIA	Ribose-5-Phosphate Isomerase A
RPE	Ribulose-5-Phosphate 3-Epimerase
G6P	Glucose 6-phosphate
G1,6 BP	Glucose-1,6-bisphosphate
N-Acetyl-GA	N-Acetyl-Glutamic Acid
IMP	Inosine Monophosphate
AMP	Adenosine Monophosphate
GMP	Guanosine Monophosphate
UMP	Uridine Monophosphate
DTDP	Deoxythymidine Diphosphate
ADP	Adenosine Diphosphate

## References

[1] Hoshino Y. (2018). Updating the markers for oocyte quality evaluation: Intracellular temperature as a new index. Reprod. Med. Biol..

[2] van der Reest J., Nardini Cecchino G., Haigis M.C., Kordowitzki P. (2021). Mitochondria: Their relevance during oocyte ageing. Ageing Res. Rev..

[3] Sha Q.-Q., Zheng W., Wu Y.-W., Li S., Guo L., Zhang S., Lin G., Ou X.-H., Fan H.-Y. (2020). Dynamics and clinical relevance of maternal mRNA clearance during the oocyte-to-embryo transition in humans. Nat. Commun..

[4] Baldini G.M., Lot D., Malvasi A., Laganà A.S., Vimercati A., Dellino M., Cicinelli E., Baldini D., Trojano G. (2024). Abnormalities of Oocyte Maturation: Mechanisms and Implications. Int. J. Mol. Sci..

[5] Conti M., Franciosi F. (2018). Acquisition of oocyte competence to develop as an embryo: Integrated nuclear and cytoplasmic events. Hum. Reprod. Update.

[6] Duarte-da-Fonseca Dias S., Palmeira-de-Oliveira A., Rolo J., Gomes-Ruivo P., Hélio Oliani A., Palmeira-de-Oliveira R., Martinez-de-Oliveira J., Pinto-de-Andrade L. (2022). Parameters influencing the maturation of bovine oocyte: A review. Anim. Prod. Sci..

[7] Hashimoto S., Gamage U., Inoue Y., Iwata H., Morimoto Y. (2025). Nicotinamide mononucleotide boosts the development of bovine oocyte by enhancing mitochondrial function and reducing chromosome lagging. Sci. Rep..

[8] Yang Y.-H., Wen R., Yang N., Zhang T.-N., Liu C.-F. (2023). Roles of protein post-translational modifications in glucose and lipid metabolism: Mechanisms and perspectives. Mol. Med..

[9] Francés-Herrero E., Lorenzo-Rebenaque L., Casto-Rebollo C., Vicente J.S., Sebastian-Leon P., Bueno-Fernandez C., Rodríguez-Eguren A., Gómez-Álvarez M., Faus A., Diaz-Gimeno P. (2024). Oviductal extracellular matrix hydrogels enhance in vitro culture of rabbit embryos and reduce deficiencies during assisted reproductive technologies. Sci. Rep..

[10] Gualtieri R., De Gregorio V., Candela A., Travaglione A., Genovese V., Barbato V., Talevi R. (2024). In Vitro Culture of Mammalian Embryos: Is There Room for Improvement?. Cells.

[11] Xiong Y.-Y., Zhu H.-Y., Shi R.-J., Wu Y.-F., Fan Y., Jin L. (2024). Regulation of glucose metabolism: Effects on oocyte, preimplantation embryo, assisted reproductive technology and embryonic stem cell. Heliyon.

[12] Pan B., Qin J., Du K., Zhang L., Jia G., Ye J., Liang Q., Yang Q., Zhou G. Integrated ultrasensitive metabolomics and single-cell transcriptomics identify crucial regulators of sheep oocyte maturation and early embryo development in vitro. J. Adv. Res..

[13] Huang J., Chen P., Jia L., Li T., Yang X., Liang Q., Zeng Y., Liu J., Wu T., Hu W. (2023). Multi-Omics Analysis Reveals Translational Landscapes and Regulations in Mouse and Human Oocyte Aging. Adv. Sci..

[14] Li L., Zhu S., Shu W., Guo Y., Guan Y., Zeng J., Wang H., Han L., Zhang J., Liu X. (2020). Characterization of Metabolic Patterns in Mouse Oocytes during Meiotic Maturation. Mol. Cell.

[15] Gao M., Chen M., Chen Q., Zhu S., Wang H., Yang W., Wang X., Wang Q., Gu L. (2023). Integration of parallel metabolomics and transcriptomics reveals metabolic patterns in porcine oocytes during maturation. Front. Endocrinol..

[16] Zhang X., Ge J., Wang Y., Chen M., Guo X., Zhu S., Wang H., Wang Q. (2024). Integrative Omics Reveals the Metabolic Patterns During Oocyte Growth. Mol. Cell. Proteom..

[17] Ma Y., Zhang W., Gao M., Li J., Wang Q., Chen M., Gu L. (2024). Combined analysis of temporal metabolomics and transcriptomics reveals the metabolic patterns in goat oocytes during maturation. Theriogenology.

[18] Zhao J., Yao K., Yu H., Zhang L., Xu Y., Chen L., Sun Z., Zhu Y., Zhang C., Qian Y. (2021). Metabolic remodelling during early mouse embryo development. Nat. Metab..

[19] Graf A., Krebs S., Zakhartchenko V., Schwalb B., Blum H., Wolf E. (2014). Fine mapping of genome activation in bovine embryos by RNA sequencing. Proc. Natl. Acad. Sci. USA.

[20] Liu T., Qu J., Tian M., Yang R., Song X., Li R., Yan J., Qiao J. (2022). Lipid Metabolic Process Involved in Oocyte Maturation During Folliculogenesis. Front. Cell Dev. Biol..

[21] Khan R., Jiang X., Hameed U., Shi Q. (2021). Role of Lipid Metabolism and Signaling in Mammalian Oocyte Maturation, Quality, and Acquisition of Competence. Front. Cell Dev. Biol..

[22] de Carvalho C., Caramujo M.J. (2018). The Various Roles of Fatty Acids. Molecules.

[23] Houten S.M., Wanders R.J.A. (2010). A general introduction to the biochemistry of mitochondrial fatty acid β-oxidation. J. Inherit. Metab. Dis..

[24] Koves T.R., Ussher J.R., Noland R.C., Slentz D., Mosedale M., Ilkayeva O., Bain J., Stevens R., Dyck J.R.B., Newgard C.B. (2008). Mitochondrial Overload and Incomplete Fatty Acid Oxidation Contribute to Skeletal Muscle Insulin Resistance. Cell Metab..

[25] Vanella A., Russo A., Acquaviva R., Campisi A., Di Giacomo C., Sorrenti V., Barcellona M.L. (2000). L-Propionyl-carnitine as superoxide scavenger, antioxidant, and DNA cleavage protector. Cell Biol. Toxicol..

[26] Shen Z., Ma Y., Gao M., Gu L. (2024). Temporal metabolomics analysis reveals the metabolic patterns in goat cumulus cells during oocyte maturation. Gene.

[27] Pawlak P., Malyszka N., Szczerbal I., Kolodziejski P. (2020). Fatty acid induced lipolysis influences embryo development, gene expression and lipid droplet formation in the porcine cumulus cells. Biol. Reprod..

[28] Gaspar M.L., Hofbauer H.F., Kohlwein S.D., Henry S.A. (2011). Coordination of storage lipid synthesis and membrane biogenesis: Evidence for cross-talk between triacylglycerol metabolism and phosphatidylinositol synthesis. J. Biol. Chem..

[29] Khajeh M., Rahbarghazi R., Nouri M., Darabi M. (2017). Potential role of polyunsaturated fatty acids, with particular regard to the signaling pathways of arachidonic acid and its derivatives in the process of maturation of the oocytes: Contemporary review. Biomed. Pharmacother..

[30] Soares A.C.S., Lodde V., Barros R.G., Price C.A., Luciano A.M., Buratini J. (2017). Steroid hormones interact with natriuretic peptide C to delay nuclear maturation, to maintain oocyte–cumulus communication and to improve the quality of in vitro-produced embryos in cattle. Reprod. Fertil. Dev..

[31] Vítek L., Haluzík M. (2016). The role of bile acids in metabolic regulation. J. Endocrinol..

[32] Ticho A.L., Malhotra P., Dudeja P.K., Gill R.K., Alrefai W.A. (2019). Intestinal Absorption of Bile Acids in Health and Disease. Compr. Physiol..

[33] Nagy R.A., Hollema H., Andrei D., Jurdzinski A., Kuipers F., Hoek A., Tietge U.J.F. (2019). The Origin of Follicular Bile Acids in the Human Ovary. Am. J. Pathol..

[34] Lee S.H., Duron H.E., Chaudhuri D. (2023). Beyond the TCA cycle: New insights into mitochondrial calcium regulation of oxidative phosphorylation. Biochem. Soc. Trans..

[35] Kierans S.J., Taylor C.T. (2024). Glycolysis: A multifaceted metabolic pathway and signalling hub. J. Biol. Chem..

[36] Qiao J., Yu Z., Zhou H., Wang W., Wu H., Ye J. (2025). The Pentose Phosphate Pathway: From Mechanisms to Implications for Gastrointestinal Cancers. Int. J. Mol. Sci..

[37] Arnold P.K., Finley L.W.S. (2023). Regulation and function of the mammalian tricarboxylic acid cycle. J. Biol. Chem..

[38] Stincone A., Prigione A., Cramer T., Wamelink M.M., Campbell K., Cheung E., Olin-Sandoval V., Grüning N.M., Krüger A., Tauqeer Alam M. (2015). The return of metabolism: Biochemistry and physiology of the pentose phosphate pathway. Biol. Rev. Camb. Philos. Soc..

[39] Neumann N., Friz S., Forchhammer K. (2022). Glucose-1, 6-bisphosphate, a key metabolic regulator, is synthesized by a distinct family of α-phosphohexomutases widely distributed in prokaryotes. mBio.

[40] Varizhuk I.V., Oslovsky V.E., Solyev P.N., Drenichev M.S., Mikhailov S.N. (2022). Synthesis of α-d-Ribose 1-Phosphate and 2-Deoxy-α-d-Ribose 1-Phosphate Via Enzymatic Phosphorolysis of 7-Methylguanosine and 7-Methyldeoxyguanosine. Curr. Protoc..

[41] Gao N., Shang J., Huynh D., Manthati V.L., Arias C., Harding H.P., Kaufman R.J., Mohr I., Ron D., Falck J.R. (2011). Mannose-6-phosphate regulates destruction of lipid-linked oligosaccharides. Mol. Biol. Cell.

[42] Lay K.M., Ashizawa K., Nakada T., Tatemoto H. (2011). N-glycosylation of zona glycoproteins during meiotic maturation is involved in sperm-zona pellucida interactions of porcine oocytes. Theriogenology.

[43] Sakaguchi Y., Uzuhashi R., Iwata H., Monji Y., Kuwayama T. (2010). Changes in the sperm-zona pellucida binding properties during porcine oocyte maturation. J. Mamm. Ova Res..

[44] Sutton-McDowall M.L., Gilchrist R.B., Thompson J.G. (2010). The pivotal role of glucose metabolism in determining oocyte developmental competence. Reproduction.

[45] Chen M., Yang W., Guo Y., Hou X., Zhu S., Sun H., Guo X., Chen M., Wang Q. (2023). Multi-omics reveal the metabolic patterns in mouse cumulus cells during oocyte maturation. J. Ovarian Res..

[46] Yokoo M., Kimura N., Abe H., Sato E. (2008). Influence of hyaluronan accumulation during cumulus expansion on in vitro porcine oocyte maturation. Zygote.

[47] Bresnahan D.R., Catandi G.D., Peters S.O., Maclellan L.J., Broeckling C.D., Carnevale E.M. (2024). Maturation and culture affect the metabolomic profile of oocytes and follicular cells in young and old mares. Front. Cell Dev. Biol..

[48] Lieu E.L., Nguyen T., Rhyne S., Kim J. (2020). Amino acids in cancer. Exp. Mol. Med..

[49] Egbujor M.C., Olaniyan O.T., Emeruwa C.N., Saha S., Saso L., Tucci P. (2024). An insight into role of amino acids as antioxidants via NRF2 activation. Amino Acids.

[50] Walker M.C., van der Donk W.A. (2016). The many roles of glutamate in metabolism. J. Ind. Microbiol. Biotechnol..

[51] Mullen N.J., Singh P.K. (2023). Nucleotide metabolism: A pan-cancer metabolic dependency. Nat. Rev. Cancer.

[52] An H., Wang X., Li J., Sun H., Zhu S., Ge J., Han L., Shen B., Wang Q. (2024). KAS-seq profiling captures transcription dynamics during oocyte maturation. J. Ovarian Res..

[53] Obaseki E., Adebayo D., Bandyopadhyay S., Hariri H. (2024). Lipid droplets and fatty acid-induced lipotoxicity: In a nutshell. FEBS Lett..

[54] Teixeira L., Pereira-Dutra F.S., Reis P.A., Cunha-Fernandes T., Yoshinaga M.Y., Souza-Moreira L., Souza E.K., Barreto E.A., Silva T.P., Espinheira-Silva H. (2024). Prevention of lipid droplet accumulation by DGAT1 inhibition ameliorates sepsis-induced liver injury and inflammation. JHEP Rep..

[55] Ducharme N.A., Bickel P.E. (2008). Minireview: Lipid Droplets in Lipogenesis and Lipolysis. Endocrinology.

[56] Shi M., Sirard M.A. (2022). Metabolism of fatty acids in follicular cells, oocytes, and blastocysts. Reprod. Fertil..

[57] Ishizuka Y., Nakao S., Kamisako T., Yamaga K., Nakagata N., Ishizaki H., Takeo T. (2024). In vivo fertilization improved the cryotolerance and developmental ability of vitrified-warmed rat fertilized oocytes. Sci. Rep..

[58] Pei Z., Deng K., Xu C., Zhang S. (2023). The molecular regulatory mechanisms of meiotic arrest and resumption in Oocyte development and maturation. Reprod. Biol. Endocrinol..

[59] Li R., Albertini D.F. (2013). The road to maturation: Somatic cell interaction and self-organization of the mammalian oocyte. Nat. Rev. Mol. Cell Biol..

[60] Tadros W., Lipshitz H.D. (2009). The maternal-to-zygotic transition: A play in two acts. Development.

[61] Clarke H.J. (2012). Post-transcriptional control of gene expression during mouse oogenesis. Results Probl. Cell Differ..

[62] Conti M., Kunitomi C. (2024). A genome-wide perspective of the maternal mRNA translation program during oocyte development. Semin. Cell Dev. Biol..

[63] Kang M.K., Han S.J. (2011). Post-transcriptional and post-translational regulation during mouse oocyte maturation. BMB Rep..

[64] Zhao B.S., Roundtree I.A., He C. (2017). Post-transcriptional gene regulation by mRNA modifications. Nat. Rev. Mol. Cell Biol..

[65] Anderson P., Kedersha N. (2009). RNA granules: Post-transcriptional and epigenetic modulators of gene expression. Nat. Rev. Mol. Cell Biol..

[66] Sugimura S., Matoba S., Hashiyada Y., Aikawa Y., Ohtake M., Matsuda H., Kobayashi S., Konishi K., Imai K. (2012). Oxidative phosphorylation-linked respiration in individual bovine oocytes. J. Reprod. Dev..

[67] Schieber M., Chandel N.S. (2014). ROS function in redox signaling and oxidative stress. Curr. Biol..

[68] Hong Y., Boiti A., Vallone D., Foulkes N.S. (2024). Reactive Oxygen Species Signaling and Oxidative Stress: Transcriptional Regulation and Evolution. Antioxidants.

[69] Khan A.U.H., Allende-Vega N., Gitenay D., Garaude J., Vo D.-N., Belkhala S., Gerbal-Chaloin S., Gondeau C., Daujat-Chavanieu M., Delettre C. (2018). Mitochondrial Complex I activity signals antioxidant response through ERK5. Sci. Rep..

[70] Campbell K., Swann K. (2006). Ca^2+^ oscillations stimulate an ATP increase during fertilization of mouse eggs. Dev. Biol..

[71] Dalton C.M., Szabadkai G., Carroll J. (2014). Measurement of ATP in single oocytes: Impact of maturation and cumulus cells on levels and consumption. J. Cell. Physiol..

[72] Li C., Brazill J.M., Liu S., Bello C., Zhu Y., Morimoto M., Cascio L., Pauly R., Diaz-Perez Z., Malicdan M.C.V. (2017). Spermine synthase deficiency causes lysosomal dysfunction and oxidative stress in models of Snyder-Robinson syndrome. Nat. Commun..

[73] Satarker S., Wilson J., Kolathur K.K., Mudgal J., Lewis S.A., Arora D., Nampoothiri M. (2024). Spermidine as an epigenetic regulator of autophagy in neurodegenerative disorders. Eur. J. Pharmacol..

[74] Zhang Y., Bai J., Cui Z., Li Y., Gao Q., Miao Y., Xiong B. (2023). Polyamine metabolite spermidine rejuvenates oocyte quality by enhancing mitophagy during female reproductive aging. Nat. Aging.

[75] Tang H., Pang S. (2016). Proline Catabolism Modulates Innate Immunity in Caenorhabditis elegans. Cell Rep..

[76] Kowsar R., Iranshahi V.N., Sadeghi N., Riasi A., Miyamoto A. (2018). Urea influences amino acid turnover in bovine cumulus-oocyte complexes, cumulus cells and denuded oocytes, and affects in vitro fertilization outcome. Sci. Rep..

[77] Santos R.R., Schoevers E.J., Roelen B.A.J. (2014). Usefulness of bovine and porcine IVM/IVF models for reproductive toxicology. Reprod. Biol. Endocrinol..

[78] Luciano A.M., Sirard M.-A. (2017). Successful in vitro maturation of oocytes: A matter of follicular differentiation. Biol. Reprod..

[79] Martinez C.A., Rizos D., Rodriguez-Martinez H., Funahashi H. (2023). Oocyte-cumulus cells crosstalk: New comparative insights. Theriogenology.

[80] Zhou C.J., Wu S.N., Shen J.P., Wang D.H., Kong X.W., Lu A., Li Y.J., Zhou H.X., Zhao Y.F., Liang C.G. (2016). The beneficial effects of cumulus cells and oocyte-cumulus cell gap junctions depends on oocyte maturation and fertilization methods in mice. PeerJ.

[81] Yu H., Shi Y., Wu X., Hu B., Jin H., Kassim Y., Iqbal T., Kandil O.M., Ismail E.A., Wang H. (2025). TEAD3 and TEAD4 play overlapping role in bovine preimplantation development. Reproduction.

[82] Want E.J., O’Maille G., Smith C.A., Brandon T.R., Uritboonthai W., Qin C., Trauger S.A., Siuzdak G. (2006). Solvent-dependent metabolite distribution, clustering, and protein extraction for serum profiling with mass spectrometry. Anal. Chem..

[83] Barri T., Dragsted L.O. (2013). UPLC-ESI-QTOF/MS and multivariate data analysis for blood plasma and serum metabolomics: Effect of experimental artefacts and anticoagulant. Anal. Chim. Acta.

