# Supplementary material for: Integrated Multi-Omics Analysis Reveals Key Regulators of Bovine Oocyte Maturation
Source: Int J Mol Sci. 2025 Apr 23;26(9):3973. doi: 10.3390/ijms26093973 (PMC12071811; doi:10.3390/ijms26093973)
Supplement: Supplementary file 1 [file ijms-26-03973-s001.zip › Supplementary Figures S1-S10.pdf]

Figure S1

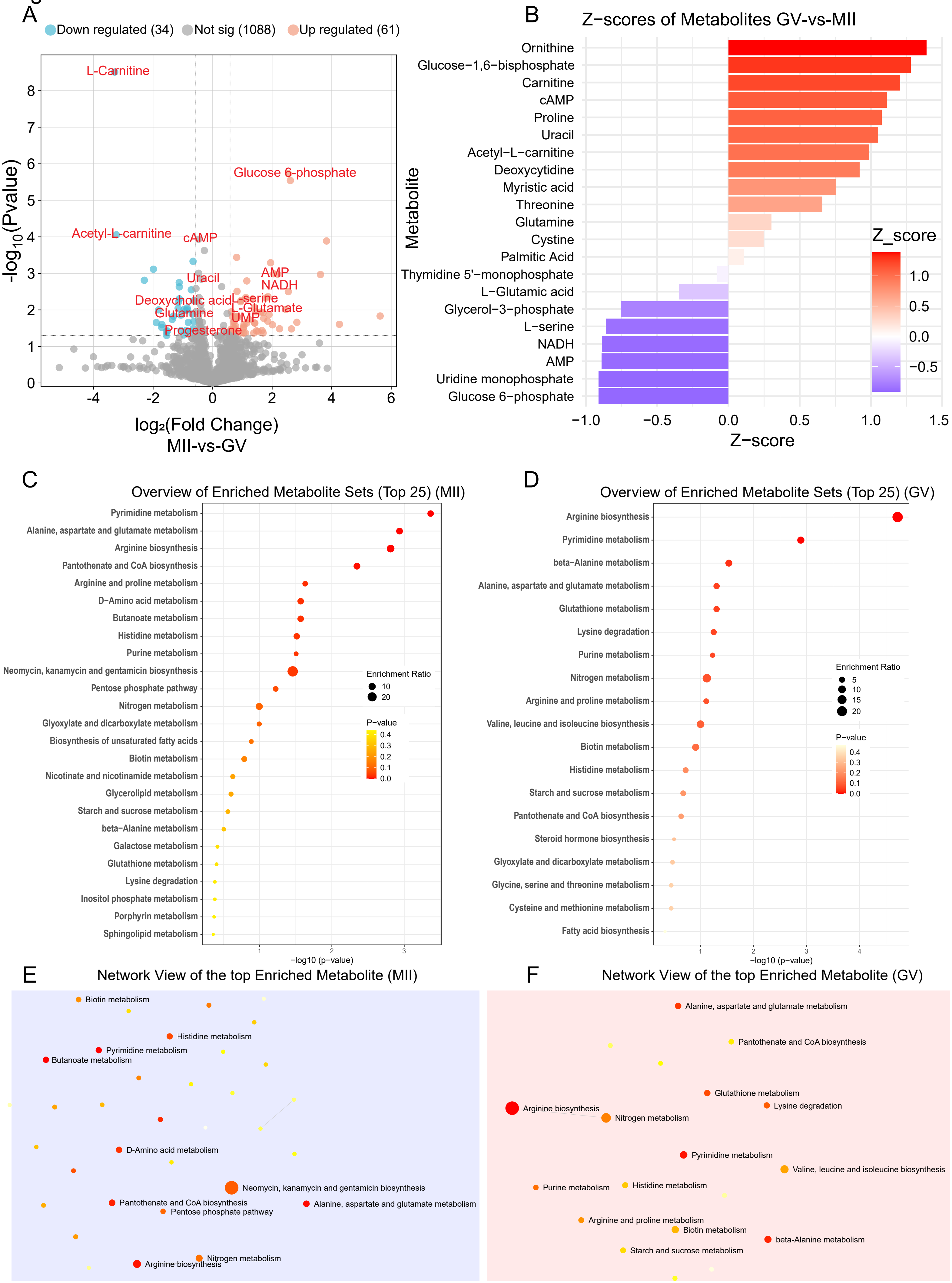
A
Down regulated (34)
Not sig (1088)
Up regulated (61)
L-Carnitine
Glucose 6-phosphate
Acetyl-L-carnitine
cAMP
Uracil
AMP
NADH
Deoxycholic acid
L-serine
Glutamine
L-Glutamate
UMP
Progesterone
-log10(Pvalue)
log2(Fold Change)
MII-vs-GV
B
Z−scores of Metabolites GV-vs-MII
Ornithine
Glucose−1,6−bisphosphate
Carnitine
cAMP
Proline
Uracil
Acetyl−L−carnitine
Deoxycytidine
Myristic acid
Threonine
Glutamine
Cystine
Palmitic Acid
Thymidine 5'−monophosphate
L−Glutamic acid
Glycerol−3−phosphate
L−serine
NADH
AMP
Uridine monophosphate
Glucose 6−phosphate
Metabolite
Z−score
Z_score
C
Overview of Enriched Metabolite Sets (Top 25) (MII)
Pyrimidine metabolism
Alanine, aspartate and glutamate metabolism
Arginine biosynthesis
Pantothenate and CoA biosynthesis
Arginine and proline metabolism
D−Amino acid metabolism
Butanoate metabolism
Histidine metabolism
Purine metabolism
Neomycin, kanamycin and gentamicin biosynthesis
Pentose phosphate pathway
Nitrogen metabolism
Glyoxylate and dicarboxylate metabolism
Biosynthesis of unsaturated fatty acids
Biotin metabolism
Nicotinate and nicotinamide metabolism
Glycerolipid metabolism
Starch and sucrose metabolism
beta−Alanine metabolism
Galactose metabolism
Glutathione metabolism
Lysine degradation
Inositol phosphate metabolism
Porphyrin metabolism
Sphingolipid metabolism
Enrichment Ratio
P−value
−log10 (p−value)
D
Overview of Enriched Metabolite Sets (Top 25) (GV)
Arginine biosynthesis
Pyrimidine metabolism
beta−Alanine metabolism
Alanine, aspartate and glutamate metabolism
Glutathione metabolism
Lysine degradation
Purine metabolism
Nitrogen metabolism
Arginine and proline metabolism
Valine, leucine and isoleucine biosynthesis
Biotin metabolism
Histidine metabolism
Starch and sucrose metabolism
Pantothenate and CoA biosynthesis
Steroid hormone biosynthesis
Glyoxylate and dicarboxylate metabolism
Glycine, serine and threonine metabolism
Cysteine and methionine metabolism
Fatty acid biosynthesis
Enrichment Ratio
P−value
−log10 (p−value)
E
Network View of the top Enriched Metabolite (MII)
Biotin metabolism
Histidine metabolism
Pyrimidine metabolism
Butanoate metabolism
D-Amino acid metabolism
Neomycin, kanamycin and gentamicin biosynthesis
Pantothenate and CoA biosynthesis
Pentose phosphate pathway
Alanine, aspartate and glutamate metabolism
Nitrogen metabolism
Arginine biosynthesis
F
Network View of the top Enriched Metabolite (GV)
Alanine, aspartate and glutamate metabolism
Pantothenate and CoA biosynthesis
Glutathione metabolism
Lysine degradation
Arginine biosynthesis
Nitrogen metabolism
Pyrimidine metabolism
Valine, leucine and isoleucine biosynthesis
Purine metabolism
Histidine metabolism
Arginine and proline metabolism
Biotin metabolism
beta-Alanine metabolism
Starch and sucrose metabolism

Figure S2

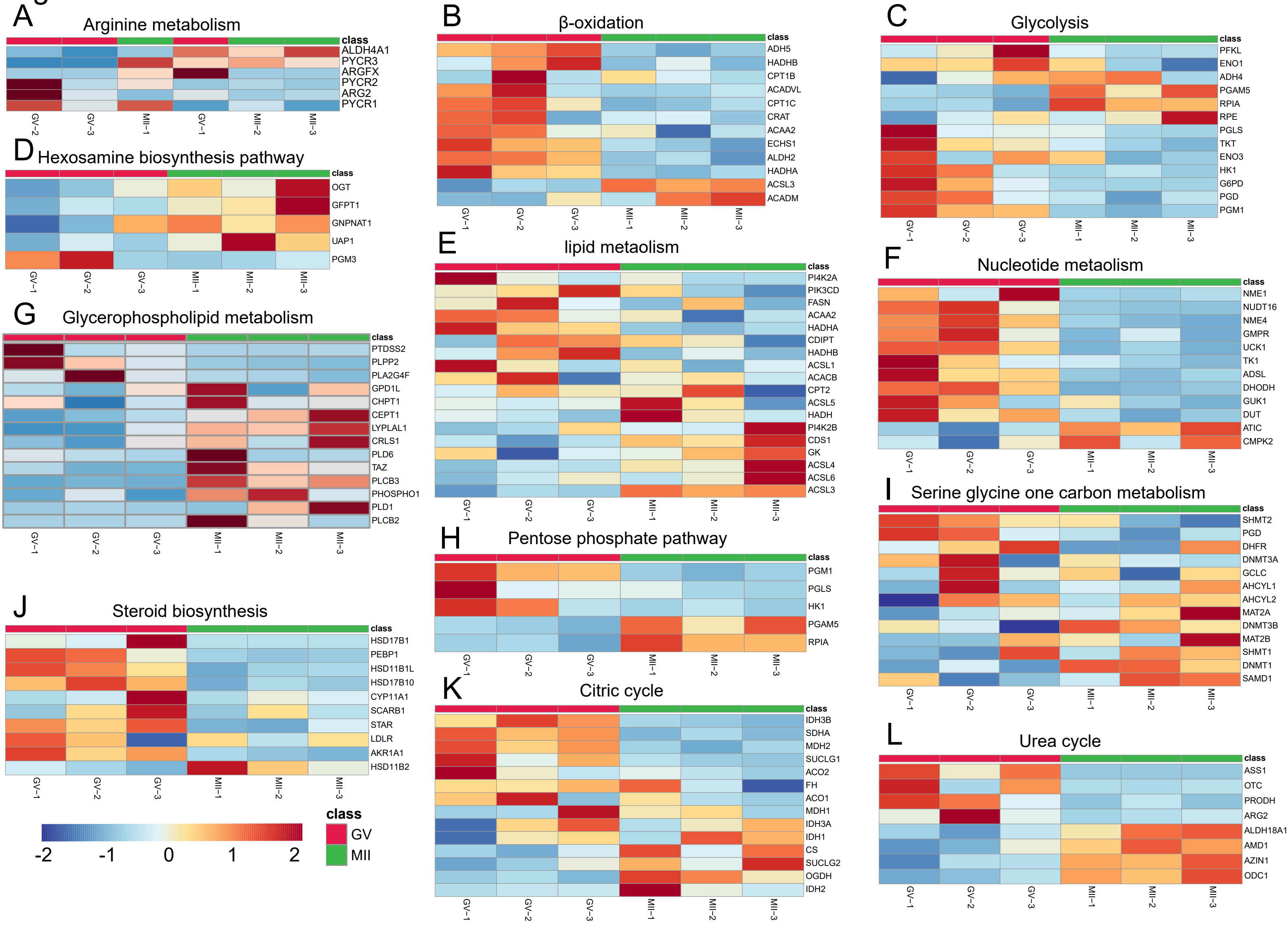
A
Arginine metabolism
class
ALDH4A1
PYCR3
ARGFX
PYCR2
ARG2
PYCR1
GV−2
GV−3
MII−1
GV−1
MII−2
MII−3
B
β-oxidation
class
ADH5
HADHB
CPT1B
ACADVL
CPT1C
CRAT
ACAA2
ECHS1
ALDH2
HADHA
ACSL3
ACADM
GV−1
GV−2
GV−3
MII−1
MII−2
MII−3
C
Glycolysis
class
PFKL
ENO1
ADH4
PGAM5
RPIA
RPE
PGLS
TKT
ENO3
HK1
G6PD
PGD
PGM1
GV−1
GV−2
GV−3
MII−1
MII−2
MII−3
D
Hexosamine biosynthesis pathway
class
OGT
GFPT1
GNPNAT1
UAP1
PGM3
GV−1
GV−2
GV−3
MII−1
MII−2
MII−3
E
lipid metaolism
class
PI4K2A
PIK3CD
FASN
ACAA2
HADHA
CDIPT
HADHB
ACSL1
ACACB
CPT2
ACSL5
HADH
PI4K2B
CDS1
GK
ACSL4
ACSL6
ACSL3
GV−1
GV−2
GV−3
MII−1
MII−2
MII−3
F
Nucleotide metaolism
class
NME1
NUDT16
NME4
GMPR
UCK1
TK1
ADSL
DHODH
GUK1
DUT
ATIC
CMPK2
GV−1
GV−2
GV−3
MII−1
MII−2
MII−3
G
Glycerophospholipid metabolism
class
PTDSS2
PLPP2
PLA2G4F
GPD1L
CHPT1
CEPT1
LYPLAL1
CRLS1
PLD6
TAZ
PLCB3
PHOSPHO1
PLD1
PLCB2
GV−1
GV−2
GV−3
MII−1
MII−2
MII−3
H
Pentose phosphate pathway
class
PGM1
PGLS
HK1
PGAM5
RPIA
GV−1
GV−2
GV−3
MII−1
MII−2
MII−3
I
Serine glycine one carbon metabolism
class
SHMT2
PGD
DHFR
DNMT3A
GCLC
AHCYL1
AHCYL2
MAT2A
DNMT3B
MAT2B
SHMT1
DNMT1
SAMD1
GV−1
GV−2
GV−3
MII−1
MII−2
MII−3
J
Steroid biosynthesis
class
HSD17B1
PEBP1
HSD11B1L
HSD17B10
CYP11A1
SCARB1
STAR
LDLR
AKR1A1
HSD11B2
GV−1
GV−2
GV−3
MII−1
MII−2
MII−3
K
Citric cycle
class
IDH3B
SDHA
MDH2
SUCLG1
ACO2
FH
ACO1
MDH1
IDH3A
IDH1
CS
SUCLG2
OGDH
IDH2
GV−1
GV−2
GV−3
MII−1
MII−2
MII−3
L
Urea cycle
class
ASS1
OTC
PRODH
ARG2
ALDH18A1
AMD1
AZIN1
ODC1
GV−1
GV−2
GV−3
MII−1
MII−2
MII−3
-2
-1
0
1
2
class
GV
MII

Figure S3

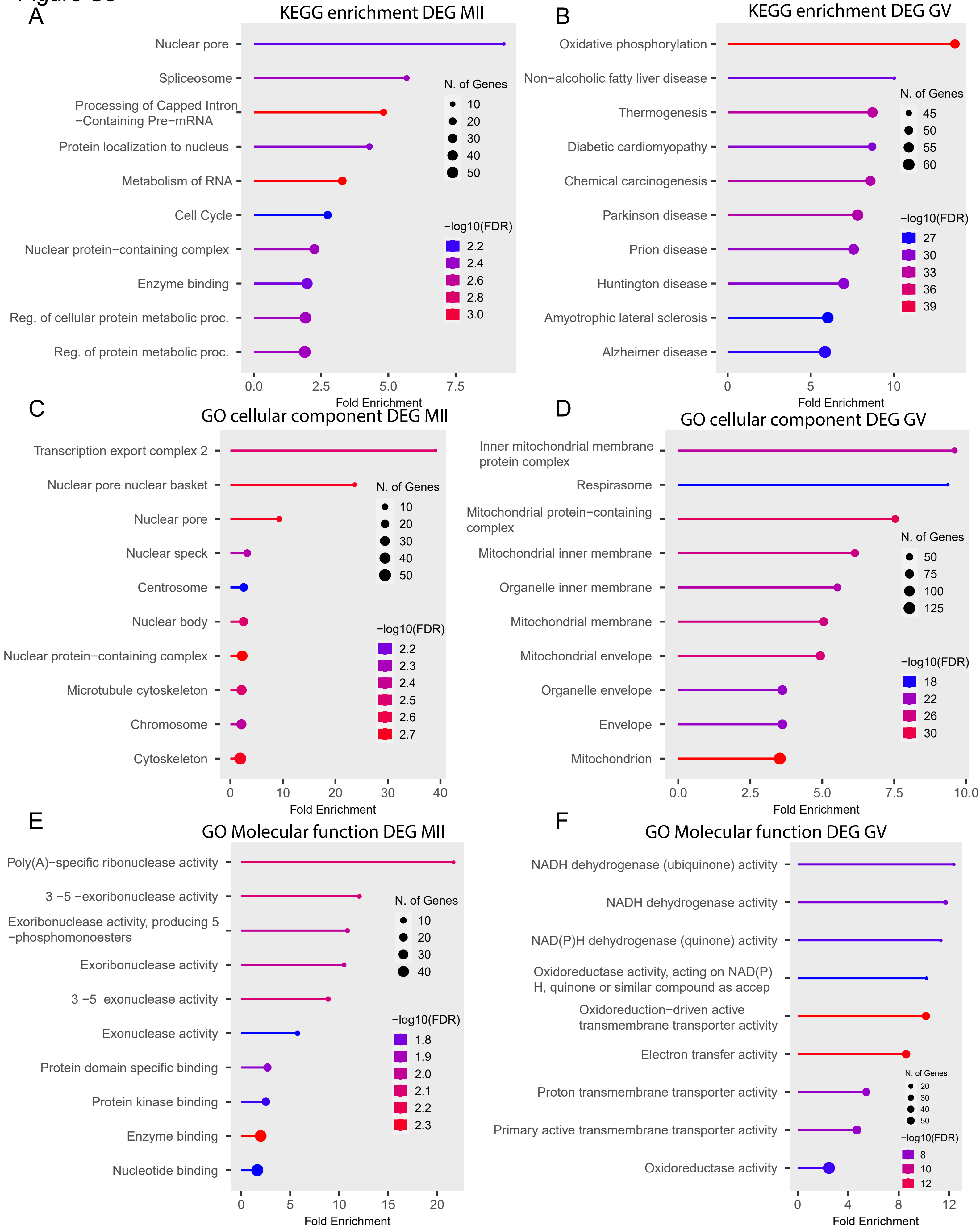
A
KEGG enrichment DEG MII
Nuclear pore
Spliceosome
Processing of Capped Intron
−Containing Pre−mRNA
Protein localization to nucleus
Metabolism of RNA
Cell Cycle
Nuclear protein−containing complex
Enzyme binding
Reg. of cellular protein metabolic proc.
Reg. of protein metabolic proc.
N. of Genes
10
20
30
40
50
−log10(FDR)
2.2
2.4
2.6
2.8
3.0
0.0
2.5
5.0
7.5
Fold Enrichment
B
KEGG enrichment DEG GV
Oxidative phosphorylation
Non−alcoholic fatty liver disease
Thermogenesis
Diabetic cardiomyopathy
Chemical carcinogenesis
Parkinson disease
Prion disease
Huntington disease
Amyotrophic lateral sclerosis
Alzheimer disease
N. of Genes
45
50
55
60
−log10(FDR)
27
30
33
36
39
0
5
10
Fold Enrichment
C
GO cellular component DEG MII
Transcription export complex 2
Nuclear pore nuclear basket
Nuclear pore
Nuclear speck
Centrosome
Nuclear body
Nuclear protein−containing complex
Microtubule cytoskeleton
Chromosome
Cytoskeleton
N. of Genes
10
20
30
40
50
−log10(FDR)
2.2
2.3
2.4
2.5
2.6
2.7
0
10
20
30
40
Fold Enrichment
D
GO cellular component DEG GV
Inner mitochondrial membrane
protein complex
Respirasome
Mitochondrial protein−containing
complex
Mitochondrial inner membrane
Organelle inner membrane
Mitochondrial membrane
Mitochondrial envelope
Organelle envelope
Envelope
Mitochondrion
N. of Genes
50
75
100
125
−log10(FDR)
18
22
26
30
0.0
2.5
5.0
7.5
10.0
Fold Enrichment
E
GO Molecular function DEG MII
Poly(A)−specific ribonuclease activity
3 −5 −exoribonuclease activity
Exoribonuclease activity, producing 5
−phosphomonoesters
Exoribonuclease activity
3 −5 exonuclease activity
Exonuclease activity
Protein domain specific binding
Protein kinase binding
Enzyme binding
Nucleotide binding
N. of Genes
10
20
30
40
−log10(FDR)
1.8
1.9
2.0
2.1
2.2
2.3
0
5
10
15
20
Fold Enrichment
F
GO Molecular function DEG GV
NADH dehydrogenase (ubiquinone) activity
NADH dehydrogenase activity
NAD(P)H dehydrogenase (quinone) activity
Oxidoreductase activity, acting on NAD(P)
H, quinone or similar compound as accep
Oxidoreduction−driven active
transmembrane transporter activity
Electron transfer activity
Proton transmembrane transporter activity
Primary active transmembrane transporter activity
Oxidoreductase activity
N. of Genes
20
30
40
50
−log10(FDR)
8
10
12
0
4
8
12
Fold Enrichment

Figure S4

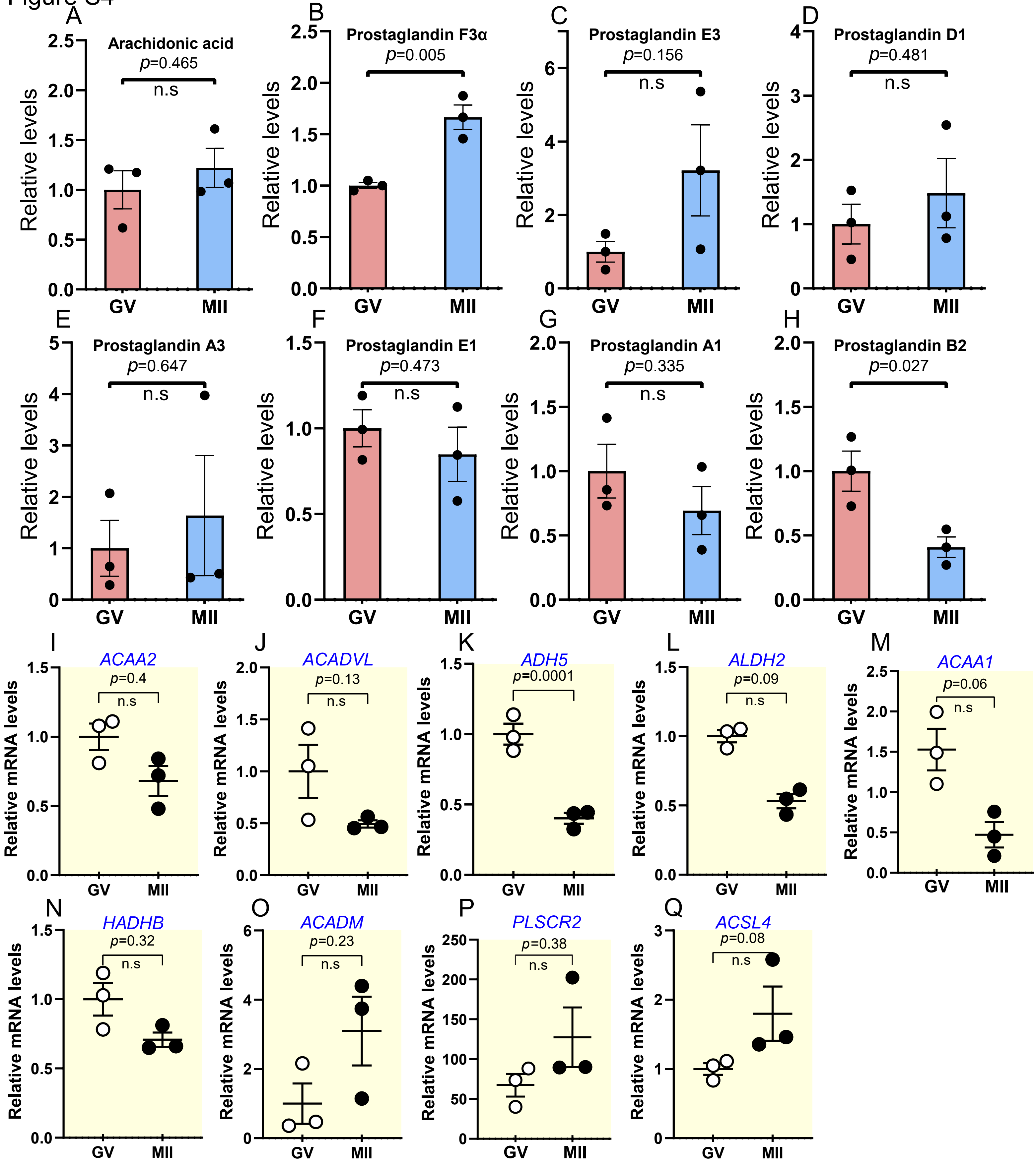

ACAA2: Acetyl-CoA Acyltransferase 2

ADH5: alcohol dehydrogenase 5 (class III)

ACAA1: Acetyl-CoA Acyltransferase 1

ACADM: Acyl-CoA Dehydrogenase Medium Chain

PLSCR2: Phospholipid Scramblase 2

ACADVL: very long-chain acyl-CoA dehydrogenase

ALDH2: Aldehyde Dehydrogenase 2 Family Member

HADHB: hydroxyacyl-CoA dehydrogenase trifunctional-multienzyme complex subunit beta

ACSL4: Acyl-CoA Synthetase Long-Chain Family Member 4

Figure S5

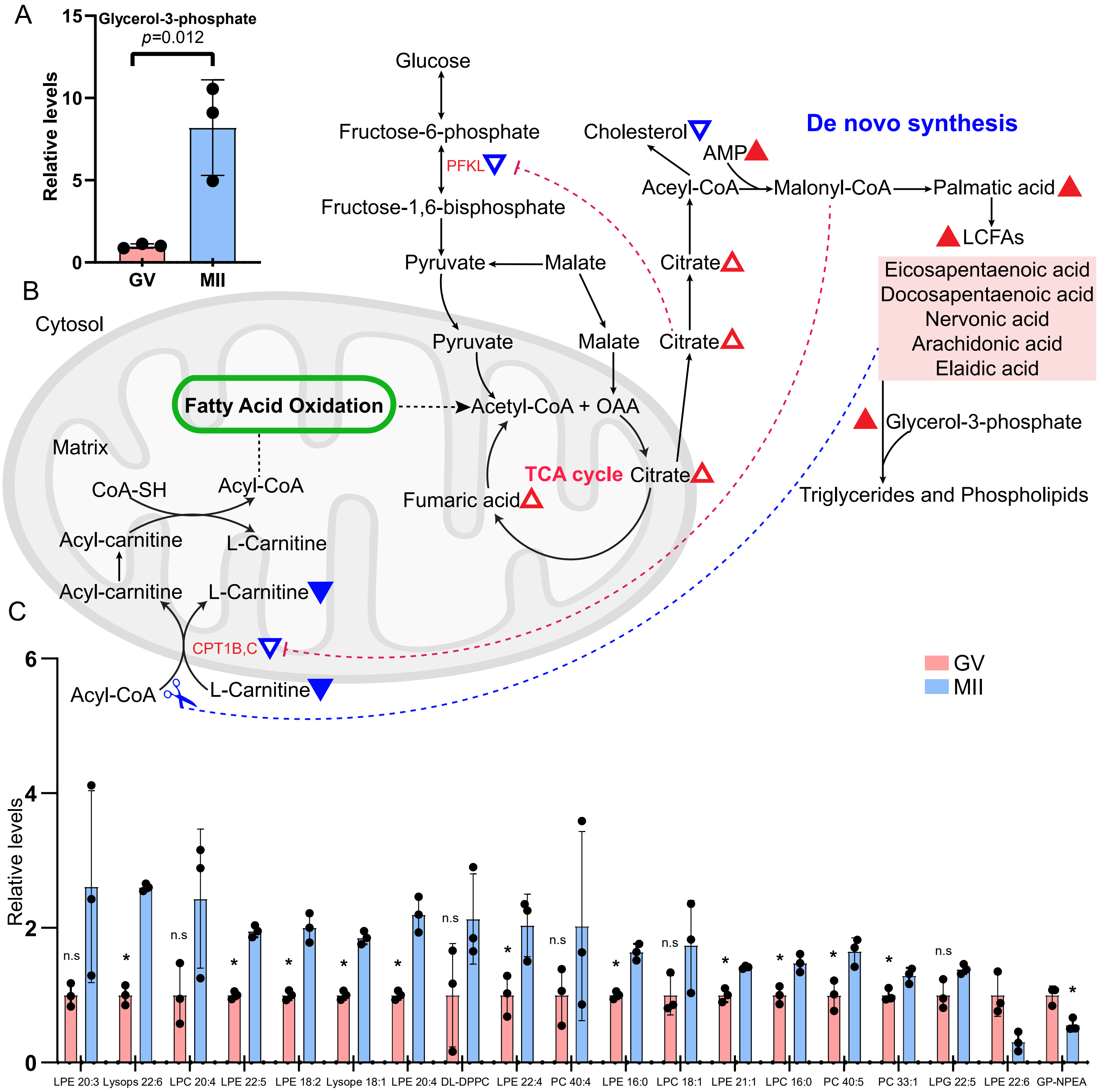

A
Glycerol-3-phosphate
p=0.012
Relative levels
15
10
5
0
GV
MII
B
Cytosol
Matrix
Glucose
Fructose-6-phosphate
PFKL
Fructose-1,6-bisphosphate
Pyruvate
Malate
Pyruvate
Malate
Cholesterol
AMP
Aceyl-CoA
Malonyl-CoA
Palmatic acid
LCFAs
De novo synthesis
Citrate
Citrate
Eicosapentaenoic acid
Docosapentaenoic acid
Nervonic acid
Arachidonic acid
Elaidic acid
Glycerol-3-phosphate
Triglycerides and Phospholipids
Fatty Acid Oxidation
Acetyl-CoA + OAA
TCA cycle
Citrate
Fumaric acid
CoA-SH
Acyl-CoA
Acyl-carnitine
L-Carnitine
Acyl-carnitine
L-Carnitine
CPT1B,C
Acyl-CoA
L-Carnitine
C
6
4
2
0
Relative levels
GV
MII
n.s
*
LPE 20:3
Lysops 22:6
LPC 20:4
LPE 22:5
LPE 18:2
Lysope 18:1
LPE 20:4
DL-DPPC
LPE 22:4
PC 40:4
LPE 16:0
LPC 18:1
LPE 21:1
LPC 16:0
PC 40:5
PC 33:1
LPG 22:5
LPE 22:6
GP-NPEA

Figure S6

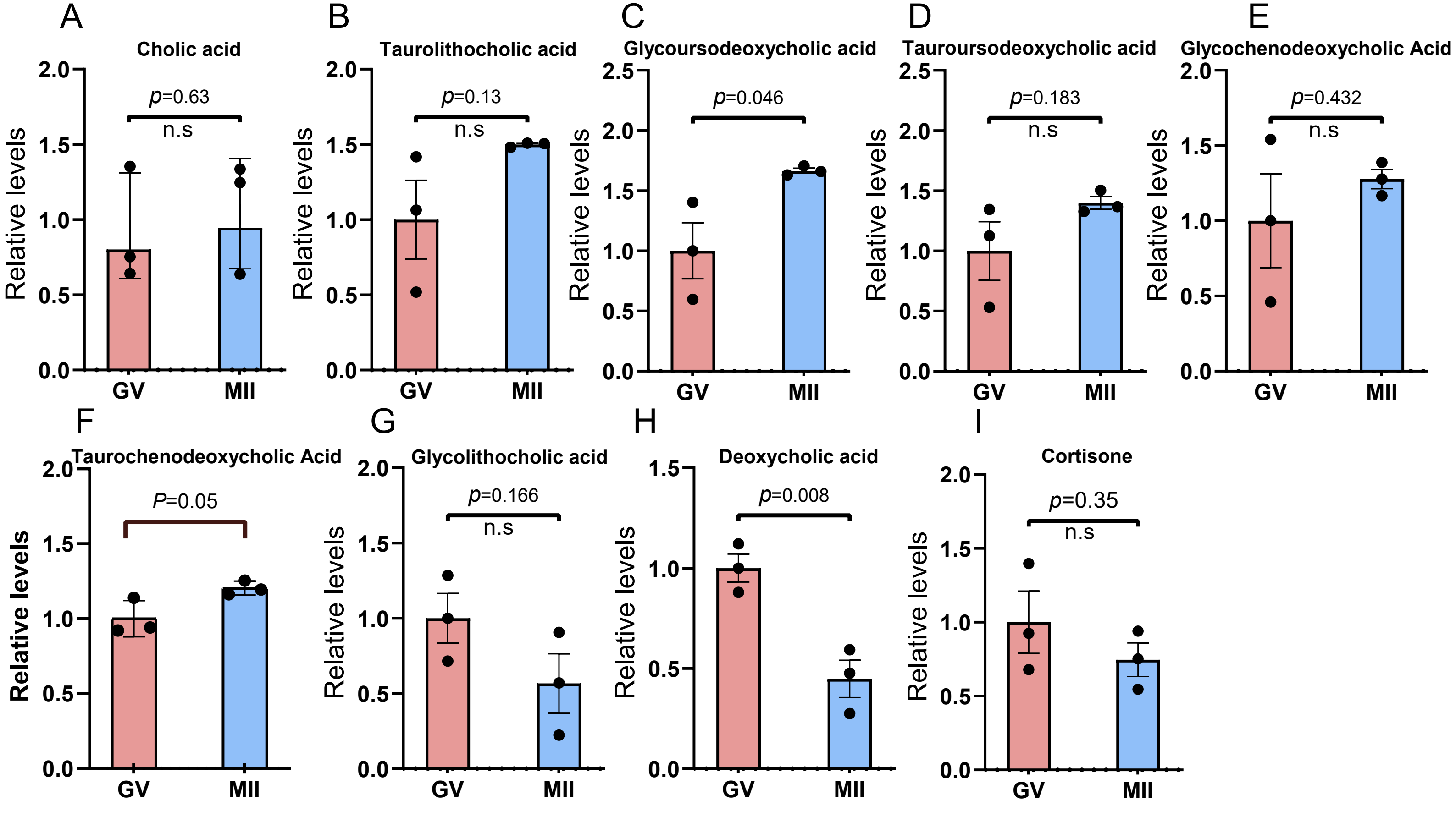
A
Cholic acid
p=0.63
n.s
B
Taurolithocholic acid
p=0.13
n.s
C
Glycoursodeoxycholic acid
p=0.046
D
Tauroursodeoxycholic acid
p=0.183
n.s
E
Glycochenodeoxycholic Acid
p=0.432
n.s
F
Taurochenodeoxycholic Acid
P=0.05
G
Glycolithocholic acid
p=0.166
n.s
H
Deoxycholic acid
p=0.008
I
Cortisone
p=0.35
n.s
Relative levels
GV
MII

Figure S7

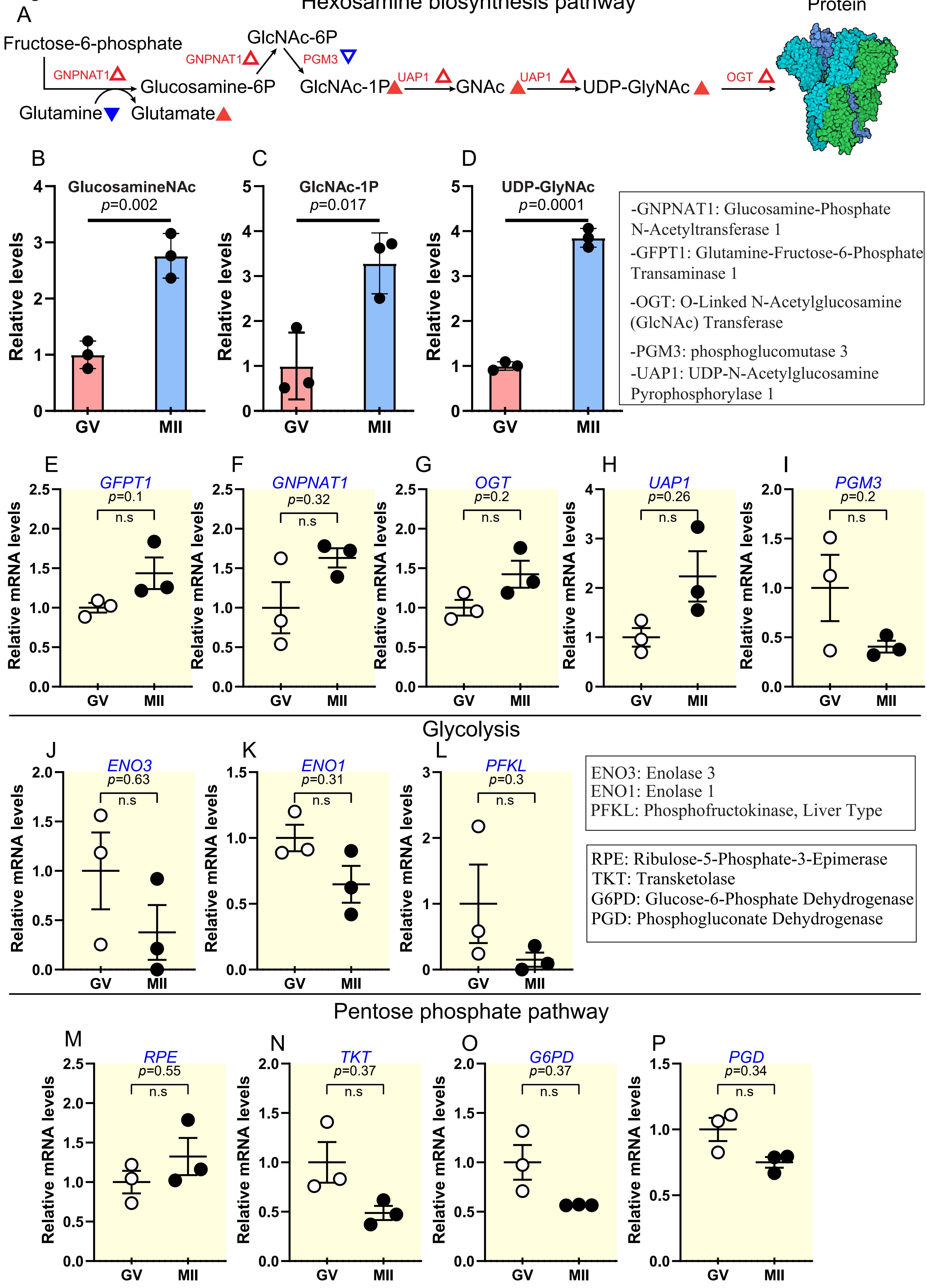
A
Hexosamine biosynthesis pathway
Protein
Fructose-6-phosphate
GNPNAT1
Glutamine
Glutamate
Glucosamine-6P
GlcNAc-6P
PGM3
GlcNAc-1P
UAP1
GNAc
UAP1
UDP-GlyNAc
OGT
B
GlucosamineNAc
p=0.002
C
GlcNAc-1P
p=0.017
D
UDP-GlyNAc
p=0.0001
Relative levels
GV
MII
-GNPNAT1: Glucosamine-Phosphate N-Acetyltransferase 1
-GFPT1: Glutamine-Fructose-6-Phosphate Transaminase 1
-OGT: O-Linked N-Acetylglucosamine (GlcNAc) Transferase
-PGM3: phosphoglucomutase 3
-UAP1: UDP-N-Acetylglucosamine Pyrophosphorylase 1
E
GFPT1
p=0.1
F
GNPNAT1
p=0.32
G
OGT
p=0.2
H
UAP1
p=0.26
I
PGM3
p=0.2
n.s
Relative mRNA levels
Glycolysis
J
ENO3
p=0.63
K
ENO1
p=0.31
L
PFKL
p=0.3
ENO3: Enolase 3
ENO1: Enolase 1
PFKL: Phosphofructokinase, Liver Type
RPE: Ribulose-5-Phosphate-3-Epimerase
TKT: Transketolase
G6PD: Glucose-6-Phosphate Dehydrogenase
PGD: Phosphogluconate Dehydrogenase
Pentose phosphate pathway
M
RPE
p=0.55
N
TKT
p=0.37
O
G6PD
p=0.37
P
PGD
p=0.34

Figure S8

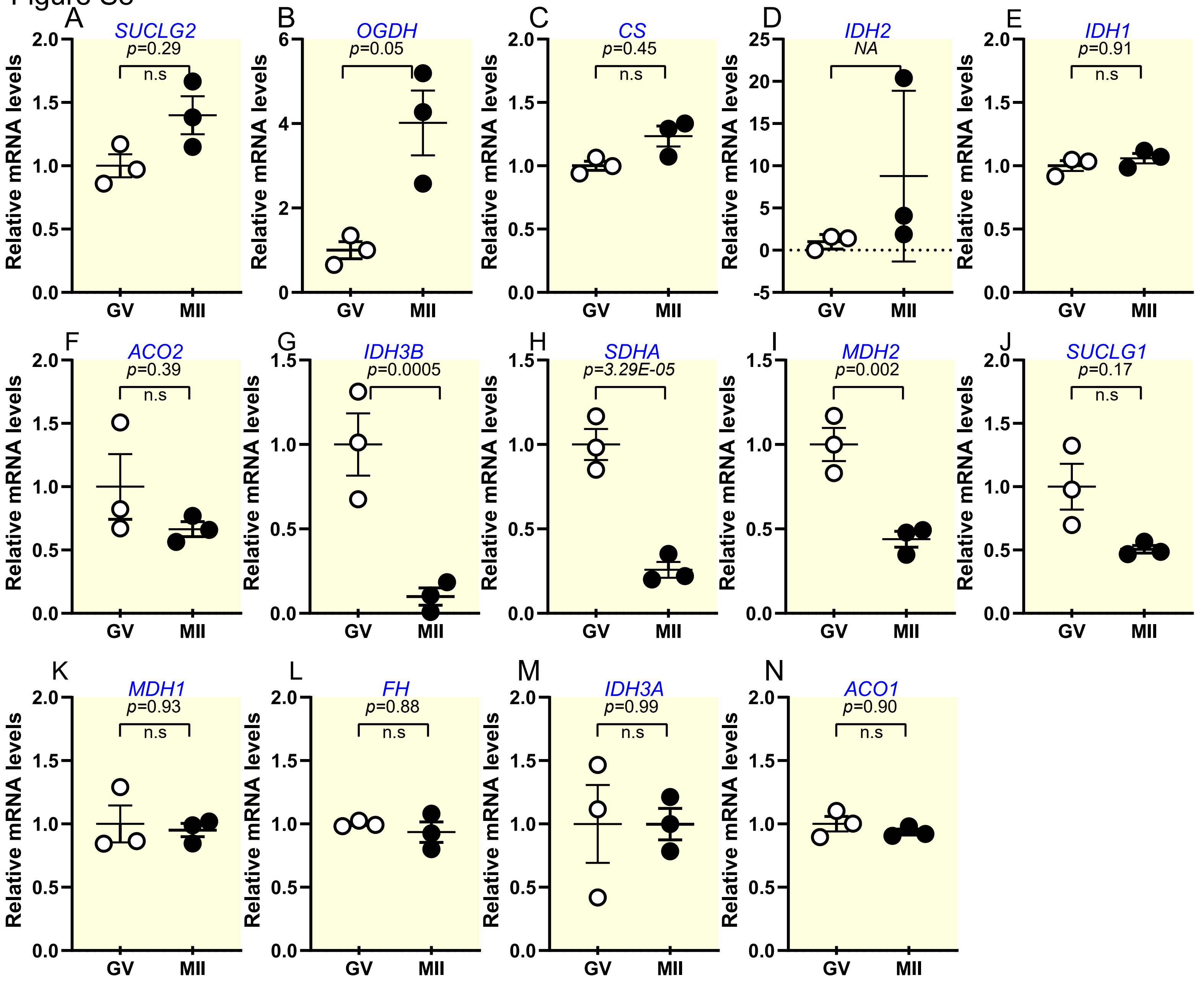

SUCLG2: Succinate-CoA Ligase GDP/ADP-Forming Subunit Gamma 2
OGDH: Oxoglutarate Dehydrogenase (α-KG Dehydrogenase)
IDH3A: Isocitrate Dehydrogenase 3 (NAD+) Subunit Alpha
IDH1: Isocitrate Dehydrogenase 1 (NADP+), cytosolic
SUCLG1: Succinate-CoA Ligase GDP/ADP-Forming Subunit Gamma 1
IDH3B: Isocitrate Dehydrogenase 3 (NAD+) Subunit Beta
SDHA: Succinate Dehydrogenase Complex Flavoprotein Subunit A
CS: Citrate Synthase
IDH2: Isocitrate Dehydrogenase 2 (NADP+)
MDH1: Malate Dehydrogenase 1, cytosolic
MDH2: Malate Dehydrogenase 2, mitochondrial
ACO2: Aconitase 2, mitochondrial
ACO1: Aconitase 1
FH: Fumarate Hydratase

Figure S9

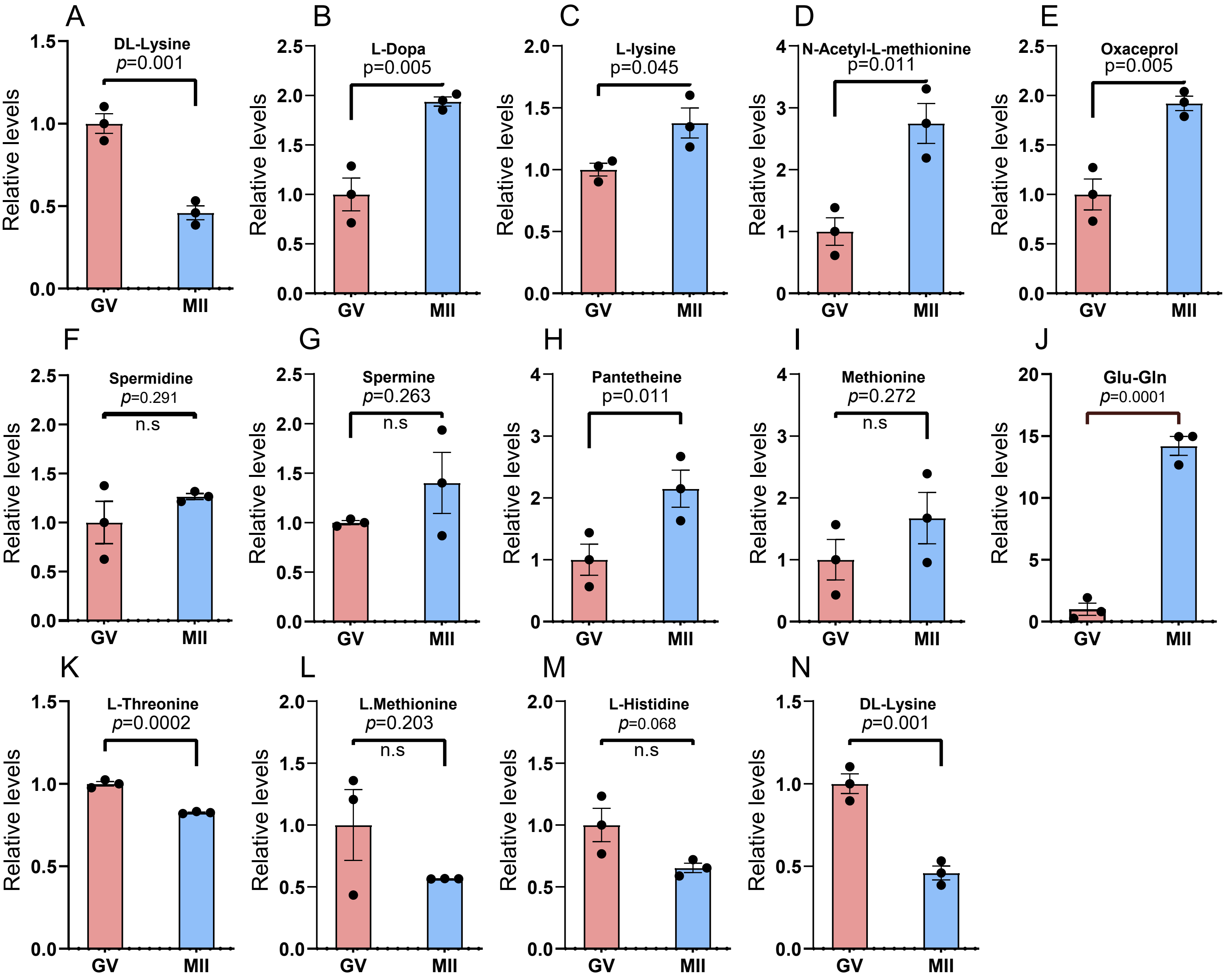

A
DL-Lysine
p=0.001
B
L-Dopa
p=0.005
C
L-lysine
p=0.045
D
N-Acetyl-L-methionine
p=0.011
E
Oxaceprol
p=0.005
F
Spermidine
p=0.291
n.s
G
Spermine
p=0.263
n.s
H
Pantetheine
p=0.011
I
Methionine
p=0.272
n.s
J
Glu-Gln
p=0.0001
K
L-Threonine
p=0.0002
L
L.Methionine
p=0.203
n.s
M
L-Histidine
p=0.068
n.s
N
DL-Lysine
p=0.001
Relative levels
GV
MII

Figure S10

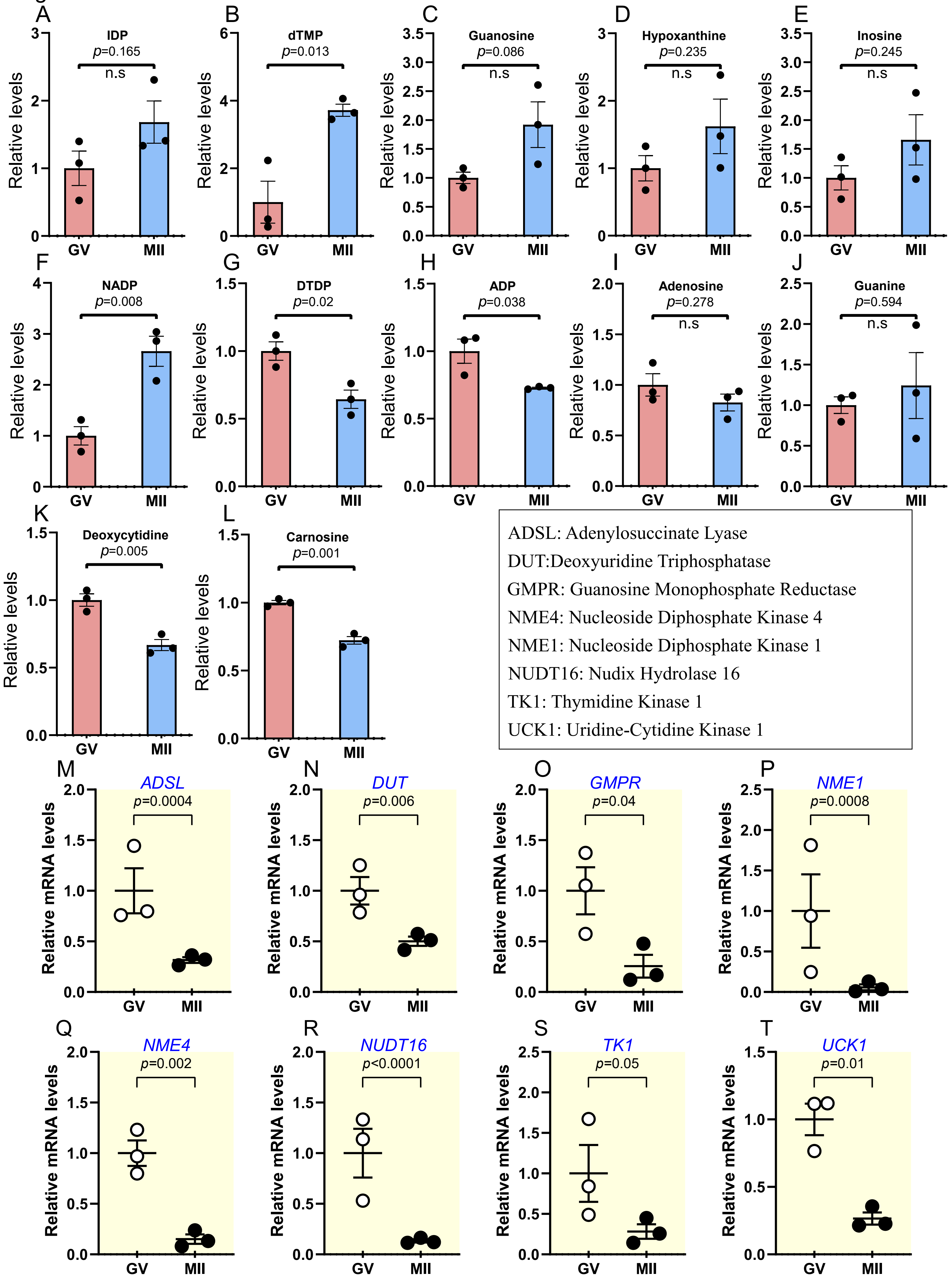
A
IDP
p=0.165
n.s
B
dTMP
p=0.013
C
Guanosine
p=0.086
n.s
D
Hypoxanthine
p=0.235
n.s
E
Inosine
p=0.245
n.s
F
NADP
p=0.008
G
DTDP
p=0.02
H
ADP
p=0.038
I
Adenosine
p=0.278
n.s
J
Guanine
p=0.594
n.s
K
Deoxycytidine
p=0.005
L
Carnosine
p=0.001
Relative levels
GV
MII
ADSL: Adenylosuccinate Lyase
DUT:Deoxyuridine Triphosphatase
GMPR: Guanosine Monophosphate Reductase
NME4: Nucleoside Diphosphate Kinase 4
NME1: Nucleoside Diphosphate Kinase 1
NUDT16: Nudix Hydrolase 16
TK1: Thymidine Kinase 1
UCK1: Uridine-Cytidine Kinase 1
M
ADSL
p=0.0004
N
DUT
p=0.006
O
GMPR
p=0.04
P
NME1
p=0.0008
Q
NME4
p=0.002
R
NUDT16
p<0.0001
S
TK1
p=0.05
T
UCK1
p=0.01
Relative mRNA levels
